# A systematic review on exercise and training-based interventions for freezing of gait in Parkinson’s disease

**DOI:** 10.1038/s41531-021-00224-4

**Published:** 2021-09-10

**Authors:** Moran Gilat, Pieter Ginis, Demi Zoetewei, Joni De Vleeschhauwer, Femke Hulzinga, Nicholas D’Cruz, Alice Nieuwboer

**Affiliations:** KU Leuven, Department of Rehabilitation Sciences, Neurorehabilitation Research Group (eNRGy), Leuven, Belgium

**Keywords:** Parkinson's disease, Rehabilitation

## Abstract

Freezing of gait (FOG) in Parkinson’s disease (PD) causes severe patient burden despite pharmacological management. Exercise and training are therefore advocated as important adjunct therapies. In this meta-analysis, we assess the existing evidence for such interventions to reduce FOG, and further examine which type of training helps the restoration of gait function in particular. The primary meta-analysis across 41 studies and 1838 patients revealed a favorable moderate effect size (ES = −0.37) of various training modalities for reducing subjective FOG-severity (*p* < 0.00001), though several interventions were not directly aimed at FOG and some included non-freezers. However, exercise and training also proved beneficial in a secondary analysis on freezers only (ES = −0.32, *p* = 0.007). We further revealed that dedicated training aimed at reducing FOG episodes (ES = −0.24) or ameliorating the underlying correlates of FOG (ES = −0.40) was moderately effective (*p* < 0.01), while generic exercises were not (ES = −0.14, *p* = 0.12). Relevantly, no retention effects were seen after cessation of training (ES = −0.08, *p* = 0.36). This review thereby supports the implementation of targeted training as a treatment for FOG with the need for long-term engagement.

## Introduction

Freezing of gait (FOG) is a very disabling paroxysmal symptom affecting over half of people with Parkinson’s disease (PD)^1^. During FOG, patients experience a sudden episodic inability to take an effective step while walking, turning, or initiating gait, leading to a marked reduction or complete cessation in forward progression of the feet despite the intention to walk^1^. FOG episodes are characterized by trembling of the knees, short shuffling steps or complete akinesia, and usually last 1–2 s, although longer periods can occur^1,2^. FOG drastically increases the risk and fear of falling^3^.

The most frequent trigger for FOG is turning^4–6^, while other common drivers include performing cognitive challenges while walking (i.e., dual-tasking)^5,7^, overcoming environmental challenges, such as negotiating doorways^8,9^, approaching destinations^6^, and reduced visual input, such as when walking in the dark^10^. Greater anxiety has also been related to worse FOG^11^.

Like most other symptoms of PD, FOG is more pronounced with advanced disease and when OFF dopaminergic medication^12^. A more specific explanation related to FOG is that episodic shortages of dorsal striatal dopamine in PD will lead to transient epochs of over-activity in the striatal output nuclei (i.e., globus pallidus internus and substantia nigra pars reticulata) and bursts of GABAergic inhibitory projections to the motor thalamus and brainstem locomotor regions inducing gait breakdown^13,14^. Dysfunctional cortical and cerebellar projections to the basal ganglia and brainstem locomotor region may exacerbate the neural manifestation of FOG^15,16^.

**Fig. 1:**
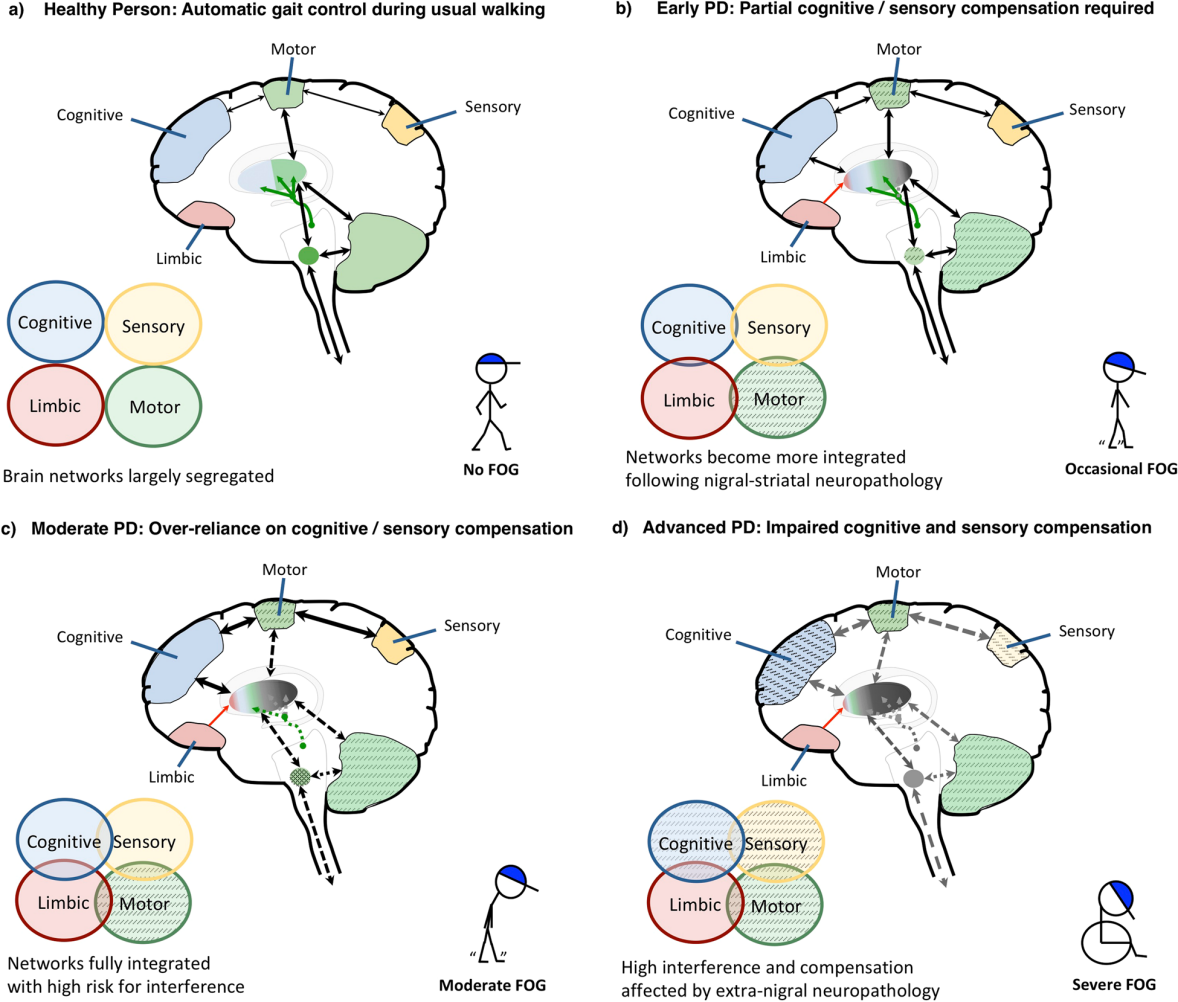
Theoretical model of gait control in healthy persons and PD patients with FOG. **a** In healthy individuals, gait automaticity is achieved via processing across dorsal cortico-striatal-thalamo-cerebellar-brainstem neural circuits. Segregation of the motor circuit from other, i.e., sensory, limbic, and cognitive circuits, allows consecutive processing of multiple inputs without interference, ensuring normal gait automaticity. Note, however, that gait is not always automatically controlled. Attentional control will be called upon in gait during complex circumstances. The need for attentional gait control increases with older age and more so in pwPD. **b** Following substantial degeneration of nigral-striatal dopaminergic neurons (indicated by gray coloring in the dorsal striatum) already prominent in early PD, gait automaticity becomes impaired. External sensory input and cognitive control come on to maintain gait control (as indicated by the increase in black arrows, modeling greater inter-circuit connectivity). Occasional FOG occurs when processing demands exceed the combined capacity of motor and compensatory circuits. Limbic input to the striatum (indicated by red arrow) may increase interference and exacerbate FOG. **c** The progression of nigral-striatal neurodegeneration inherent to moderate PD heavily affects processing across the motor circuit (indicated by dotted arrows), increasing the dependency on compensatory circuits. The risk for interference during gait becomes higher, resulting in regular episodes of FOG. **d** In the advanced stages, extra-nigral neuropathology starts to affect processing across the compensatory circuits (indicated by the gray dotted arrows), resulting in severe gait disability and frequent FOG.

Figure 1 shows a simplified representation of the brain circuits involved in maintaining gait in healthy adults and PD. As stated above, the pathophysiology of FOG likely involves both localized primary dysfunction of the dorsal motor circuit and a breakdown across compensatory networks^13,14,17–19^.

Figure 1a displays the dorsal cortico-basal ganglia and cerebellar motor circuit implicated in the acquisition and execution of automated movements, which in healthy individuals frees up attentional and sensory resources that can then be used to process secondary task demands during walking in usual conditions^20–22^. Specifically, although all these networks interact to some degree when processing varying gait demands, cognitive and sensory input is less essential to maintain a steady gait in healthy subjects.

Figure 1b illustrates that as a result of impaired processing in the dorsal striatum, PD patients become increasingly reliant on external sensory input and cognitive control for their gait, switching to goal-directed behavior^23^. An increased reliance on goal-directed gait control is also evident in healthy older adults^24^, but is more pronounced in pwPD, especially in those who experience FOG^25^. Relatively early in the disease, processing across the affected striatum and compensatory networks is capable of controlling gait reasonably well^13,17^, although changes in gait metrics can already be detected, such as slower gait speed and increased stride-time variability^25^. At this stage, FOG episodes may occur occasionally and particularly during complex situations whereby the demand for gait control exceeds the combined processing capacity of the motor-cognitive circuits, such as during turning^4^. Aberrant inputs from the limbic circuitry, in particular during high threat conditions^11^, may put further strain on striatal processing and thereby exacerbate FOG^13,16,26^. Dopamine replacement therapy and deep brain stimulation (DBS) of the subthalamic nucleus (STN) may at this point still facilitate processing across the dorsal motor circuit, allowing partially automated gait control and thereby reducing the risk for FOG^27,28^.

Unfortunately, over time, the inevitable progression of nigral-striatal degeneration will heavily affect processing across the dorsal motor circuit, as shown in Fig. 1c, likely increasing the dependency on compensatory modulation^17,18^. As a result, the higher competition for neural recourses will amplify the risk of transient interference, and as such FOG will emerge more regularly^13,14,17^. Medications and DBS may reduce the frequency of FOG, but are at this point no longer fully adequate for clinical management^19,29^.

In the more advanced stages of PD, as shown by Fig. 1d, extra-nigral neuropathology is thought to influence the participation of compensatory gait circuits, which together with the heavily affected motor circuit results in severe gait disability and regular FOG^18^. At this stage, when compensatory gait control starts to fail, dopamine replacement therapies and DBS no longer suffice to treat FOG^29,30^.

Clearly, FOG is a highly complex symptom with both motor and non-motor correlates that may be amendable with behavioral therapy. Different types of behavioral interventions can be designed that can safely target FOG at one or multiple of three levels, namely: (1) the circumvention of imminent episodes, such as via the application of cognitive strategies and/or cueing to induce compensatory gait control; (2) reducing the impact of provocative factors, such as by training pwPD on how to deal with the different triggers of FOG, and; (3) boost compensatory gait control and/or reduce gait interference by targeting the underlying correlates of FOG with the aim to increase resilience against the occurrence of FOG (Fig. 1). However, questions remain on how strong the evidence is for training- and exercise-based interventions to reduce FOG and how a more systematic approach can be facilitated.

Four recent systematic reviews focusing on various forms of exercise incorporated FOG only as a secondary outcome^31–34^. Rutz and Benninger conducted a systematic review on physical therapy specifically for FOG, but included other gait disturbances as well^35^. Moreover, no meta-analysis was performed to support their conclusions^35^.

Two additional systematic reviews were published which addressed whether non-pharmacological interventions had an effect on FOG as a primary outcome. The first summarized evidence on varying therapeutics including non-invasive brain stimulation, and as such did not focus specifically on exercise- or training-based trials^36^. Due to the extensive clinical heterogeneity of the intervention types, a meta-analysis was not feasible. The review also included open-label studies, as well as cross-sectional studies without a training component lasting >1 day, questioning whether the outcomes reflected clinically relevant interventions^36^.

The second systematic review selected only RCT studies that represented physiotherapy interventions with FOG as a primary outcome, irrespective of whether it was reported as such in the selected studies^37^. Other strengths were that the authors compared the effects of physical therapy separately for studies with active (i.e., sham intervention) and those with passive (i.e., no treatment) control groups. The outcomes of this meta-analysis revealed that physical therapy improved subjective FOG as compared to both active (*n* = 10, *Z* = 3.90, *p* < 0.001) and passive control groups (*n* = 9, *Z* = 3.42, *p* < 0.001). Long-term retention effects remained significant across eight studies comparing physical therapy to an active control intervention (*Z* = 3.89, *p* < 0.001), in particular following action observation (n = 4, p = 0.002), but not cueing (*n* = 2, *p* = 0.78). However, Cosentino et al. did not include all types of exercise interventions (e.g., dance and tai-chi), resulting in a total of 19 included studies. Hence, subgroup analyses were low in power and should be interpreted with caution. Finally, the effects of physiotherapy-based interventions that only enrolled PD patients who experience FOG, i.e., freezers, were not examined^37^.

Given the complexity of FOG and the involvement of various compensatory circuits and primary motor networks, the present systematic review aims to assess the effects of a broad spectrum of exercise- and training-based interventions on FOG. As exercise compliance and effort is not self-evident in PD^38^, this review aims to underpin evidence-based choices to engage in exercise according to patients’ preferences. As per Cosentino et al., we will also differentiate between studies with contrasts to either active (i.e., sham) or passive (i.e., no treatment) control groups, and determine the long-term retention effects^37^. Most importantly, to address the specificity of the evidence for FOG, we will investigate studies that enrolled freezers only, given that the more severe disease profiles of freezers could affect effect sizes.

As a secondary aim, we will assess which intervention type may benefit FOG most based on a novel conceptual framework. For this purpose, interventions are split into three subgroups based on their relevance to FOG (Box 1), i.e., A. FOG-specific, B. FOG-relevant, and C. generic exercise. The latter category was included to test the hypothesis that even exercises that are conventionally offered to the wider population for their general health benefits could also benefit FOG in pwPD, as sometimes postulated by the authors of these studies. Critically, the generic exercise interventions did not specifically target FOG as the primary outcome.

Taken together, this systematic review with meta-analysis intends to take a major step forward in determining the evidence for reducing the severity of FOG with various training modes. Based on a critical appraisal of the existing literature, we will provide a comprehensive overview of the benefit of rehabilitation to counter this highly debilitating symptom of PD. As such, we will contribute to a framework for methodical clinical reasoning on how to implement training-interventions over the course of the disease.

Box 1 Three conceptual categories of training interventions as based on their relevance to FOG

1. FOG-specific
  Exercise or training-based interventions aimed directly at alleviating imminent FOG episodes or better prepare patients for upcoming FOG while the interventions are applied, and possibly in times beyond. This includes mixed intervention studies, of which at least one training component is directly aimed at reducing FOG episodes or circumventing FOG-provoking circumstances. Examples are: cueing offered to help patients overcome FOG episodes; action-observation training strategies designed to relieve FOG in FOG-provoking situations; and fall-prevention training including strategies to overcome imminent FOG episodes, such as through the use of cueing. FOG was assessed as a primary or other outcome in these studies.
2. FOG-relevant
  Exercise or training-based interventions aimed at training the motor- and/or non-motor correlates of FOG with the aim to reduce the severity or amount of FOG following the intervention, but not aimed at the immediate alleviation of imminent FOG episodes or circumventing FOG-provoking circumstances while the intervention was applied. Examples are cognitive training; cognitive-motor dual-task training; balance training; curved treadmill training; regular treadmill training with cueing that was aimed at improving gait parameters other than FOG; and obstacle avoidance training. FOG was assessed as a primary or other outcome in these studies.
3. Generic exercises
  Conventional physical therapy or generic exercise interventions that are also frequently offered to healthy older individuals to improve physical- and/or mental fitness and other benefits, irrespective of their possible potential to also benefit FOG. Examples are different types of dancing; yoga; physiotherapy not aimed at FOG; aquatic training; Tai-chi; gait training; muscle-power training, and; music therapy. FOG was not assessed as a primary outcome in these studies.

## Systematic search

### Preregistration

The protocol for this systematic review with meta-analysis was prospectively registered and published online on PROSPERO (CRD:42019123882) and can be found in Supplementary Note 1.

### Search strategy

Following recent recommendations^39^, we searched for literature in the following combination of databases: PubMed, EMBASE, MEDLINE Ovid, Web of Science core collection, and Google Scholar from conception until the 3rd of August 2020. The following combination of search terms was used: Parkinson disease; AND (Freezing OR festination, shuffling); AND (gait OR walking); AND (rehabilitation OR training, exercise, physiotherapy, physical therapy, non-pharmacological, behavioral, cueing, cues, feedback, action observation, dual-task, virtual reality, exergaming, cognitive, auditory, visual, executive, sensory, proprioceptive, imagery, treadmill, wearables, balance, dance, tango, tai chi, strength). The full search criteria are provided in Supplementary Note 1.

### Inclusion/exclusion criteria

Literature was selected based on the following a priori inclusion criteria: (i) prospectively collected empirical evidence of any kind on the effect of an exercise or training-based intervention (including cognitive training) of at least 2 days (in order to exclude pre/post studies of a single training session) with a randomized-controlled design; (ii) FOG severity being assessed as an outcome measure; (iii) written in any language and without date restrictions; (iv) article published in a peer-reviewed scientific journal; and (v) evidence based on human participants with a clinical diagnosis of idiopathic PD.

First, non-duplicate titles were screened according to two exclusion criteria: (i) title clearly indicates that the study is a review of the literature with- or without meta-analysis; (ii) title clearly indicates the study is not based on human participants (e.g., animal- or in-vitro studies). The remaining abstracts and full-texts were subsequently screened according to the following exclusion criteria: (i) review of the literature with- or without meta-analysis; (ii) no empirical data; (iii) not based on human participants; (iv) no peer-review; (v) dissertation, conference abstracts or posters; (vi) no participants with idiopathic PD; (vii) no exercise- or training-based interventions (including pharmacological, surgical or other non-behavioral interventions such as non-invasive brain stimulation); (viii) less than 10 participants in total across groups; (ix) no outcome on FOG collected; (x) not randomized-controlled; (xi) FOG only assessed at baseline; (xii) only freezing during movements other than gait (e.g., freezing of the upper limbs, foot-tapping, stationary stepping).

FOG outcomes considered included measures directly related to FOG, such as freezing documented on video, freezing ratios measured with wearable sensors, freezing rated by a clinician, and self-reported FOG using diaries and FOG-related questionnaires. Proxy measures that were indirectly related to FOG, such as measures of gait, balance, falls, activities of daily living, and quality of life were not considered in the present review, because the actual relationship between FOG-severity and these outcomes is indirect and may be unclear.

### Literature selection

**Fig. 2:**
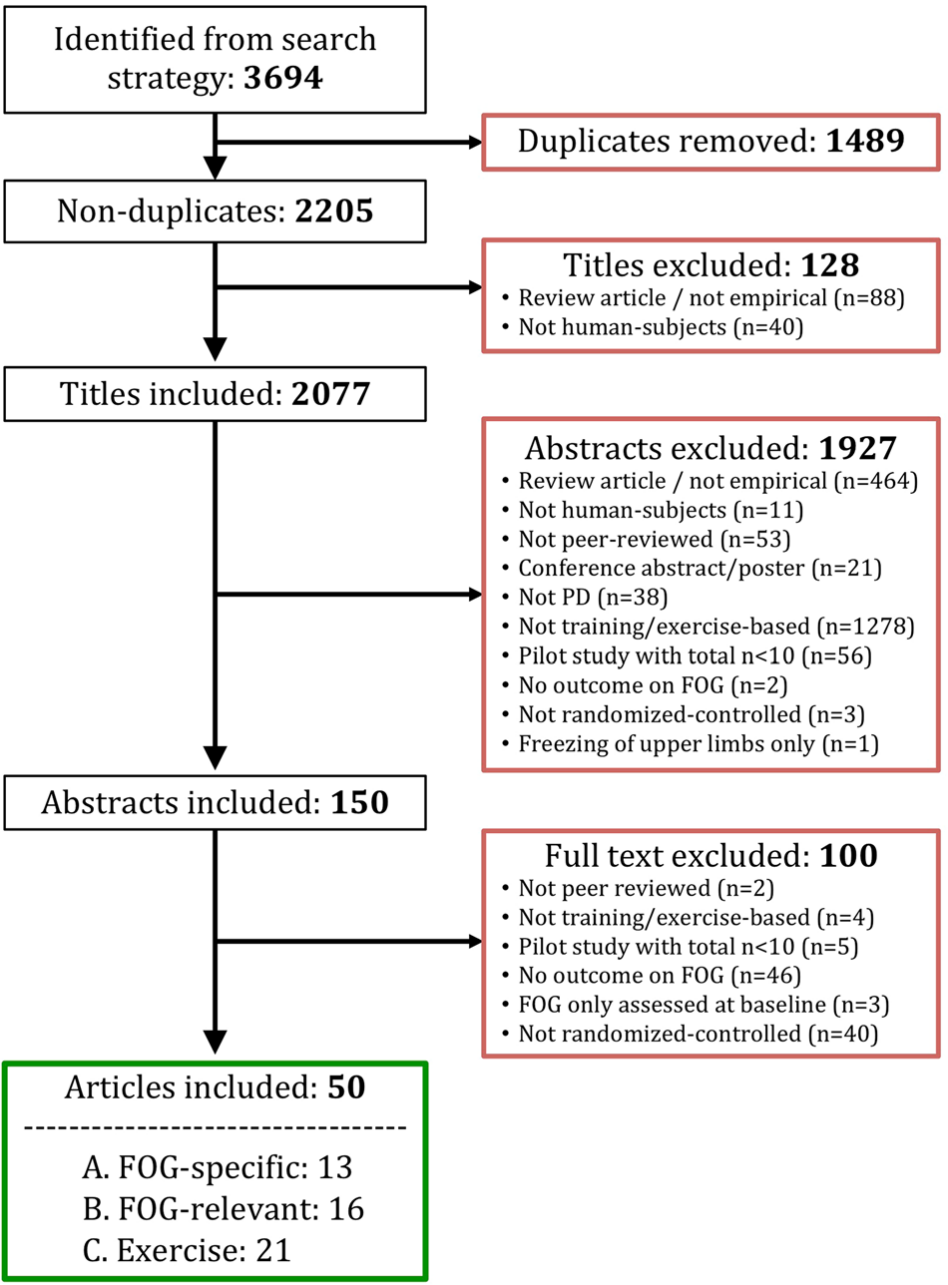
Flowchart of the systematic article selection strategy. From 3694 articles identified from the search strategy, a total of 50 articles were included for review. Red boxes indicate exclusions of articles during each of the following screening stages: duplicate removal, title, abstract, full-text. Reasons for exclusion are shown inside the red boxes. Green box indicates inclusion.

Two researchers (MG, PG) independently screened non-duplicate titles, abstracts, and full-texts according to the eligibility criteria described above (Fig. 2). In the event of a discrepancy between decisions (*n* = 2), a third moderator (ND) decided on the correct allocation. The same independent procedure was carried out to allocate studies to the conceptual categories displayed in Box 1, whereby discrepancies were resolved by another moderator (AN).

### Quality assessment

A modified version of The National Heart, Lung, and Blood Institute Quality Assessment Tool for Controlled Intervention Studies was used to assess the risk of bias within included studies. This scale evaluates the internal validity of controlled studies using 14 predefined criteria. Two assessors (MG and PG) independently scored the criteria.

## Systematic review

### Literature search results

A total of 2205 non-duplicate titles were screened, resulting in a total of 50 articles eligible for inclusion in the review (Fig. 2). A summary overview of included articles is presented in Table 1 and the full systematic overview of the included articles is presented in Supplementary Table 1.

**Table 1:** Summary overview of included studies.

Study (author, year)	Intervention 1	Intervention 2	*N* (intervention 1, 2)	%FOG (intervention 1, 2)
**CategoryA: FOG-specific**				
Ashburn (2019)^86^ & Chivers Seymour (2019)^82^ ^a^	Home-based fall prevention program	Usual care	238, 236	64, 59
Mezzarobba (2017)^44^ ^a^	AO + sonification	Sham without AO	12, 10	100, 100
Pelosin (2018)^45^	AO	Sham without AO	32, 32	100, 100
Agosta (2017)^53^	AO	Sham without AO	12, 13	100, 100
Cui (2017)^87^	PT with cueing	Sham PT without cueing	20, 20	NR, NR
Ginis (2016)^70^	Corrective feedback on gait performance	Sham gait training without corrective feedback	20, 18	70, 55.6
Canning (2015)^74^	Fall prevention exercises	Usual care	115, 116	46, 53
Martin (2015)^40^	Cueing training	Wait-list control	10, 9	100, 100
Fietzek (2014)^41^	Cueing training	Wait-list control	14, 8	100, 100
Allen (2010)^101^	Fall prevention exercises	Usual care	24, 24	NR, NR
Pelosin (2010)^46^	AO	Sham without AO	9, 9	100, 100
Nieuwboer (2007)^42^ ^a^	Cueing training at home	Wait-list control	76, 77	20.3, 20.9
**CategoryB: FOG-relevant**				
Bekkers (2020)^47^ ^a^	TT with virtual reality	Sham TT without virtual reality	34, 43	100, 100
King (2020)^43^	Agility bootcamp-cognitive program	Education and relaxation control group	23, 19	100, 100
Silva-Batista (2020)^48^	Motor-cognitive balance training	Sham traditional motor rehabilitation	17, 15	100, 100
Capecci (2019)^114^	Robot-assisted TT	Sham TT without robot	48, 48	68, 63
Clerici (2019)^49^	Motor-cognitive training + aquatic training	Sham motor-cognitive training only	27, 25	100, 100
Wróblewska (2019)^54^	Nordic walking training	Usual care	20, 20	100, 100
Schlenstedt (2018)^55^	Resistance training	Balance training	12, 8	100, 100
Walton (2018)^50^	Cognitive training specific to FOG	Sham cognitive training not specific to FOG	20, 18	100, 100
Zhu (2018)^51^	Aquatic obstacle training	Sham aquatic training without obstacles	23, 23	NR, NR
Cheng (2017)^52^	Curved walking TT	Sham trunk and upper limb exercises	12, 12	NR, NR
Santos (2017b)^100^	Slack-line training	NR	11, 11	NR, NR
Schlick (2016)^56^	TT with visual cueing not targeted at FOG	Sham TT without visual cueing	10, 10	NR, NR
King (2015)^79^	Individually-supervised (1) or group-supervised (2) agility boot camp training	Unsupervised home-based agility boot camp training (3)	21, 20, 17	46, 46, 60
Ricciardi (2015)^80^ ^b^	PT for most affected (1), or least affected body side (2)	PT for both body sides (3)	9, 9, 10	NR, NR, NR
Kadivar (2011)^83^ ^a^	Auditory cued stepping in place	Sham internally cued stepping in place	8, 8	50, 37.5
Frazzitta (2009)^57^	TT and cueing training	Sham cueing training without TT	20, 20	100, 100
**Category C: Generic exercise**				
Kalyani (2020)^58^	Dancing seated and standing	Usual care	17, 16	NR, NR
Pohl (2020)^90^	Group- and music-based Ronnie Gardiner method	Usual care	26, 20	NR, NR
Hubble (2019)^84^	Trunk exercises + fall education brochures	Sham fall education	11, 11	NR, NR
Medijainen (2019)^115^	PT including cueing not specific to FOG	Wait-list control	12, 12	NR, NR
Rocha (2018)^73^	Tango	Mixed genre dancing	10, 11	NR, NR
Sedaghati (2018)^59^	Alexander-based corrective techniques on forward flexed posture	NR	13, 13	NR, NR
Van Puymbroeck (2018)^60^	Yoga	Wait-list control	15, 12	NR, NR
Carpinella (2017)^75^	PT with biofeedback (Gamepad system)	Sham PT without biofeedback	17, 20	NR, NR
Carroll (2017)^81^	Aquatic training	Usual care	10, 8	NR, NR
Santos (2017)^61^	Progressive resistance training	Usual care	13, 15	NR, NR
Xiao (2017)^78^	Tai-Chi ball exercises	Usual care	25, 25	NR, NR
Byl (2015)^85^ ^a^	Gait training with visual and kinesthetic feedback	Sham gait training without feedback	7, 5	NR, NR
Romenets (2015)^98^	Tango	Waitlist control + pamphlet for PD exercises at home	18, 15	NR, NR
Duncan (2014)^76^ ^c^	Tango	Usual care	5, 5	NR, NR
Paul (2014)^116^	Muscle power training	Sham low-intensity exercises at home	20, 20	NR, NR
Volpe (2013)^72^	Irish set dancing	PT with cueing not specific to FOG	12, 12	NR, NR
Duncan (2012)^77^ ^a^	Tango	Usual care	26, 26	NR, NR
Reuter (2011)^68^	Nordic walking (1), or regular walking (2) training	Flexibility and relaxation training (3)	30, 30, 30	NR, NR, NR
Hackney (2009)^62^	Tango (1) or Waltz/Foxtrot (2)	Usual care (3)	14, 17, 17	57.1, 52.9, 29.4
Hackney (2007)^63^	Tango	Sham strength and flexibility exercises	9, 10	NR, NR
Pacchetti (2000)^67^	Music therapy (i.e., use of musical instruments)	PT including gait and balance training	16, 16	NR, NR

Studies are grouped per intervention category as highlighted in bold font.

*AO* Action observation, *FOG* freezing of gait, *PT* physical therapy, *TT* Treadmill training, *N* number of subjects analyzed in each group for the FOG outcome at the primary endpoint; *%FOG* Percentage of subjects that were classified as being “freezers” (i.e., people with Parkinson’s disease who experience FOG) at baseline, *NR* Not reported or values could not be computed with the information provided.

^a^FOG meta-data provided by authors upon email request.

^b^Authors contacted by email to retrieve FOG data for inclusion in the meta-analysis.

^c^Same participants as Duncan and Earhart (2012)^77^.

### Study designs

Four studies applied a crossover design^40–43^, and the rest a parallel-group design. For crossover trials, only the effects of the first intervention period (i.e., prior to crossover) were considered to minimize potentially confounding carry-over effects.

### Study Outcome

Eleven studies included FOG as a primary outcome^41,43–52^. Most of these studies were published in recent years (2017 and beyond), with the exception of Pelosin et al.^46^ and Fietzek et al.^41^. In the remaining studies, 25 specified FOG as a secondary or tertiary outcome, whilst 14 included FOG as one of the many outcomes without specifying which was considered primary^40,53–63^.

The large majority of studies assessed self-reported FOG severity using the “original” Freezing of Gait Questionnaire (FOG-Q; *n* = 37)^64^, or the New FOG-Q (NFOG-Q; *n* = 10)^65^. In 2016, the Movement Disorders Task Force deemed the FOG-Q as a recommended instrument for capturing self-perceived FOG^66^. Unlike the NFOG-Q, however, it combines the ratings of gait and FOG. Besides obtaining the FOG-Q, one study also assessed FOG severity using a diary^46^. Two other studies captured the single FOG item of either the Unified Parkinson’s Disease Rating Scale Part 2 (UPDRS-II)^53^ or the single FOG item of the UPDRS Part 3 (UPDRS-III)^41^ besides the FOG-Q. Two studies, however, assessed FOG solely using the single FOG item of the UPRDS-II^67^ or UPDRS-III^68^. Four studies also included a semi-objective outcome^69^ based on observed FOG severity ratings during the performance of a FOG-provoking walking course^41,54,55,70^. Only Fietzek et al.^41^ and Schlenstedt et al.^55^ blinded the assessors to group allocation. Two recent studies^43,48^ captured objective FOG severity using a FOG-ratio as derived from inertial measurement units during the performance of a turning on the spot task^71^. To date, only one study assessed FOG severity as the percentage of time spent with FOG as measured objectively from video recordings of standardized walking tasks and rated by independent and blinded assessors^50^.

### Study quality

The full quality assessment is presented in Table 2. All 50 studies randomly allocated participants to one of the study arms (Table 2, item 1). Kalyani et al., however, only randomized the first 75%, and manually allocated the remaining 25% of subjects based on their preference. Their trial should thus be considered pseudo-randomized^58^. A total of 36 studies reported adequate concealment of treatment allocation, mostly by using computerized randomization procedures by an independent investigator. Ten studies reported that participants were kept blinded as much as possible by not informing them of the study aims (Table 2, item 4). Thirty-three studies kept the assessors of study outcomes blinded to treatment allocation (Table 2, item 5). Two studies also blinded the treatment providers by involving physiotherapist^72^ or dance teachers^73^ to provide usual care without being told the aims of the study and group assignment of the participants. The large majority of studies (*n* = 36) reported less than 20% overall dropout. Seven studies reported more than 20% overall dropout across the interventions, whilst another six did not report the dropout rates (Table 2, item 7). The dropout rates were similar between groups in three of the seven studies (Table 2, item 8), whereas the dropout rate was higher in the control group for two studies^40,50^ and higher in the intervention group for two other studies^74,75^. Adherence rates were high (70–100%) in all of the 31 studies reporting compliance (Table 2, item 9). Only twenty studies reported to have conducted an a priori power calculation to determine the required sample size, and of these, six did not reach their recruitment target (Table 2, item 12). Adequate statistical power in the analysis can therefore only be assumed for 14 of the 50 included studies. As can be seen in Table 2, the large majority (*n* = 43) of studies had a high or unclear risk of bias on three or more items on this quality assessment scale.

**Table 2:** Quality assessment within studies per category.

	Item													
Study (author, year)	1	2	3	4	5	6	7	8	9	10	11	12	13	14
**A. FOG-specific**														
Ashburn, 2019					X		X						X	
Chivers Seymour, 2019				X								X		
Mezzarobba, 2018				X				X	X				X	X
Pelosin, 2018				X	X					X		X	X	X
Agosta, 2017				X				X	X			X	X	X
Cui, 2017			X	X	X				X	X		X	X	X
Ginis, 2016			X	X	X							X		X
Canning, 2015				X			X	X		X				
Martin, 2015			X	X	X		X		X	X		X	X	X
Fietzek, 2014				X		X			X			X	X	X
Allen, 2010				X						X			X	
Pelosin, 2010			X									X	X	X
Nieuwboer, 2007				X									X	
**B. FOG-relevant**														
Bekkers, 2020				X								X	X	X
King, 2020				X		X						X	X	X
Silva-Batista, 2020		X												X
Capecci, 2019				X										X
Clerici, 2019													X	
Wroblewska, 2019				X	X	X	X	X				X	X	X
Schlenstedt, 2018			X	X			X	X	X			X	X	X
Walton, 2018						X	X	X		X				
Zhu, 2018									X	X		X	X	X
Cheng, 2017				X										X
Santos, 2017b				X	X							X	X	X
Schlick, 2016				X	X	X			X	X		X	X	X
King, 2015				X						X				X
Ricciardi, 2015							X	X	X	X		X	X	X
Kadivar, 2011		X	X	X	X	X				X			X	X
Frazzitta, 2009		X		X	X				X	X		X	X	
**C. Generic exercise**														
Kalyani, 2020		X	X	X	X	X							X	
Pohl, 2020				X						X		X		X
Hubble, 2019				X	X		X	X		X				X
Medijainen, 2019		X	X	X						X		X	X	X
Rocha, 2018			X				X			X		X		X
Sedaghati, 2018		X	X	X	X	X	X	X	X	X		X	X	X
Van Puymbroeck, 2018				X		X			X	X		X	X	X
Carpinella, 2017				X		X	X	X	X	X		X	X	X
Carroll, 2017					X							X	X	
Santos, 2017a				X	X				X	X		X	X	X
Xiao, 2017		X	X	X	X	X	X	X	X	X		X	X	X
Byl, 2015		X	X	X	X	X			X			X	X	X
Romenets, 2015				X	X		X		X			X	X	
Duncan, 2014			X	X			/	/	X			X	X	X
Paul, 2014						X				X			X	
Volpe, 2013				X						X		X	X	X
Duncan, 2012			X	X			X			X		X		
Reuter, 2011				X								X	X	X
Hackney, 2009												X		X
Hackney, 2007		X		X								X	X	
Pachetti, 2000				X			X	X	X		X	X	X	X

Studies are grouped per intervention category as highlighted in bold font.

*Empty cell* low risk of bias, *Cell containing a cross* high or unclear risk of bias, / not Applicable. The following criteria were scored:

(1) Was the study described as randomized, a randomized trial, a randomized clinical trial, or an RCT?

(2) Was the method of randomization adequate (i.e., use of randomly generated assignment)?

(3) Was the treatment allocation concealed (so that assignments could not be predicted)?

(4) Were study participants kept blinded to the expected effects of the intervention?

(5) Were the people assessing the outcomes blinded to participants’ group assignments?

(6) Were the groups similar at baseline on important characteristics that could affect outcomes (e.g., demographics, risk factors, comorbid conditions)?

(7) Was the overall dropout rate from the study at endpoint 20% or lower of the number allocated to treatment?

(8) Was the differential dropout rate (between treatment groups) at endpoint 15 percentage points or lower?

(9) Was there high adherence (>75%) to the intervention protocols for each treatment group?

(10) Were other interventions avoided or similar in the groups (e.g., similar background treatments)?

(11) Were outcomes assessed using valid and reliable measures, implemented consistently across all study participants?

(12) Did the authors report that the sample size was sufficiently large to be able to detect a difference in the main outcome between groups with at least 80% power?

(13) Were outcomes reported or subgroups analyzed pre-specified (i.e., identified before analyses were conducted)?

(14) Were all randomized participants analyzed in the group to which they were originally assigned, i.e., did they use an intention-to-treat analysis?

### Type of interventions

Table 1 presents the precise intervention types offered in each study. Eight studies trialed a dancing-based intervention, seven a cueing-based intervention, four applied action-observation training, one cognitive training, and the remaining studies investigated different types of physical therapy such as treadmill, aquatic, Nordic walking, tai-chi, balance, resistance, yoga, slack-line, curved walking, and fall prevention exercises. For the purpose of this review, the interventions were divided into three newly devised subcategories based on their relevance to FOG (Box 1). A total of 13 studies were categorized as being specifically designed to reduce FOG episodes, 16 as being designed to ameliorate the correlates underlying FOG, and 21 as being non-specific and unrelated to FOG (Table 1).

### Dosage of interventions

The duration of the interventions ranged between 2 weeks and 2 years, with a median of 8 weeks (Supplementary Table 1). The dosage (number of weeks * number of sessions per week * time per session) of supervised interventions ranged from 3 to 224 h with a median of 15 h. The total dosage, including unsupervised (i.e., home-based) sessions also ranged from 3 to 224 h, but with a median of 18 h. In studies, which did not report the unsupervised adherence rates, we assumed a 1× per week adherence of similar duration as instructed by the investigators.

### Medication status

In 38 studies, which provided training in the ON medication state, the dopaminergic medication status of participants was reported. A total of 44 studies reported the medication status of participants during the assessments. Of these, 39 studies assessed participants during the ON state, four^43,76,77,78^ in the practically defined “OFF” state, and one study^50^ assessed participants both “ON” and “OFF” dopaminergic medications on two separate testing days (Supplementary Table 1).

### Control groups

Twenty-two studies compared the effects of the intervention against a passive control group (i.e., delayed-start or usual care control group without a training intervention designed to induce any measurable training effects on the outcomes of interest). Twenty-five studies compared the intervention group against an active (e.g., sham interventions or standard physiotherapy) control group. Finally, three studies compared the effects between two or more intervention groups and were thus not included in the meta-analysis. Specifically, King et al. compared the effects of a home-based exercise program with that of an individual- as well as a group-based exercise program^79^. The intervention contained exercises aimed at the underlying correlates of FOG (category B). Ricciardi et al. compared the effects across three physiotherapy-based programs, namely one aimed at improving the worst body-side of PD, one aimed at improving the best-side of PD, and one offering standard physiotherapy. The investigators aimed to improve step asymmetry underlying FOG (category B)^80^. Finally, Rocha et al. compared Argentinian tango dancing to mixed-genre dancing (category C)^73^.

### Study populations

Characteristics of the study populations, involving 2972 patients, are detailed in Supplementary Table 1. The number of participants in each group ranged from 5 to 238, with a median number of 17 subjects in each group. Across all active groups, 61% of participants were male and 39% female and in the control groups 55% were male and 45% female. Three studies did not report the participant’s sex^43,68,78^. The mean (SD) age across all groups was 68.8 (3.8) years, across the intervention groups 68.6 (3.7) and control groups 69.0 (4.0), ranging between 60 and 81 years on group average. A total of 45 studies reported disease duration as the number of years since clinical diagnosis. The mean (SD) disease duration across all groups was 8.4 (2.3) years, across the intervention groups 8.1 (2.2) and control groups 8.7 (2.4), ranging between 3 and 13 years on group average. A total of 42 studies reported the Hoehn and Yahr stages (HY). The median HY stage across all groups was 2.5, ranging between 1.6 and 3.2 on group average. A total of 40 studies reported the scores on the motor part of the Movement Disorders Society Unified Parkinson’s Disease Rating scale (UPDRS-III) during the dopaminergic ON state. The mean (SD) UDPRS-III across all groups was 28.3 (8.0), across the intervention groups 28.2 (8.6) and control groups 28.4 (8.2), ranging between 9.2 and 51.6 on group average. Twenty-four studies reported the group averages on the Mini-Mental State Examination (MMSE) as a rapid cognitive screening test. The mean (SD) MMSE across all these groups was 27.8 (1.1), across the intervention groups 27.7 (1.0) and control groups 27.8 (1.1), ranging from 25.5 to 29.3 on group average. Only eight studies reported the Montreal Cognitive Assessment (MoCA) as a rapid screening test that is more sensitive to the cognitive deficits in PD. The mean (SD) MoCA across the groups in these studies was 25.8 (1.0). The mean (SD) MoCA of the intervention groups was 25.9 (1.0) and of the control groups 25.6 (1.1).

## Meta analysis

### Data extraction

Two researchers (MG, PG) independently extracted relevant meta-data from the included studies using a standardized score form, see Supplementary Note 1. Inconsistencies in data entries were screened for and resolved by the researchers prior to conducting the analyses. Given that the large majority of studies assessed FOG subjectively using questionnaires, only data obtained from the FOG-Q and NFOG-Q were entered into the meta-analysis. In one study, median (interquartile-range; IQR) values were reported and due to the relatively low sample size of that study (*n* = 10 and *n* = 8 per intervention) mean (SD) values could not be imputed^81^. As such, the median (IQR) values were entered into the analysis despite the likelihood of skewness in the study data. A sensitivity analysis was performed without this particular study, showing that the results of the main analysis barely changed (primary analysis: Δ*Z* = −0.06, Δ*I*^2^ = 2%), and as such, the data of this study were entered into the analyses. Ginis et al.^70^ and Bekkers et al.^47^ reported the NFOG-Q for a subgroup of PD patients who experienced FOG in their study. In the event of missing data on FOG (*n* = 9), the corresponding authors were contacted via email with the request to enter the missing items in a standardized data collection form provided. Six of the nine contacted authors provided the missing data and could be included in the meta-analysis^43,44,47,77,82,83^, while three did not reply^80,84,85^. The data of Chivers Seymour et al.^82^ are the same as Ashburn et al.^86^ and the data of Duncan and Earhart^77^ are the same as Duncan and Earhart^76^. Hence these data were only entered once. From the Chivers Seymour et al. data set^82^, only the outcomes obtained from the PD subgroup that were classified as being freezers at baseline and for whom NFOG-Q data was available at both baseline and the primary endpoint (6 months) were included in the meta-analyses (intervention *n* = 80, control *n* = 79). Data from a subsample of included studies (*n* = 15) that also completed the (N)FOG-Q at follow-up were entered into the secondary retention analysis (intervention *n* = 257, control *n* = 263), As described in the Secondary analyses outcomes section below. Walton et al.^50^, Reuter et al.^68^ and Pacchetti et al.^67^ did not collect the (N)FOG-Q and could thus not be entered into the meta-analysis.

### Data synthesis

Data from the FOG-Q and NFOG-Q were standardized and pooled using an inverse-variance random-effects meta-analysis in RevMan (v5.3). First, mean (N)FOG-Q values at baseline were compared between intervention and control groups for each study using independent sample *t*-tests. This analysis revealed that the intervention and control groups within each study had a comparable mean (N)FOG-Q scores at baseline (all *p* > 0.05), except for the study of Wroblewska et al.^54^. As such, an analysis of final measures was employed for the meta-analysis, whereby the standardized means of the post-intervention (N)FOG-Q scores were calculated and entered into the meta-analysis with 95% confidence intervals and two-sided *p*-values. In the study of Wroblewska et al.^54^, the intervention group receiving Nordic walking training started with a higher mean (SD) FOG-Q score of 13.8 (2.3) than the passive no-intervention control group (9.3 (1.8), *p* < 0.01). Therefore, we also performed sensitivity analyses after the removal of this particular study. In another study, two intervention groups were compared against a control group^62^. As such, the mean FOG-Q scores of both intervention groups were entered into the Revman calculator and their combined standardized mean score was compared against the control group. For all analyses, heterogeneity between the studies in effect measures was explored using the *χ*^2^ and *I*^2^ statistics, whereby a significant *χ*^2^ statistic (*p* < 0.05) and/or an *I*^2^ value >50% was considered representative of substantial heterogeneity. In the event of substantial heterogeneity, a leave-one-out sensitivity analysis was conducted. Funnel plots were computed to evaluate potential bias. Studies were considered outliers if their effect estimates fell outside the 95% confidence interval of the pooled effect estimates, visualized as dotted lines in the Funnel plots. These are presented in Supplementary Data 1.

### Primary analysis outcome

**Fig. 3:**
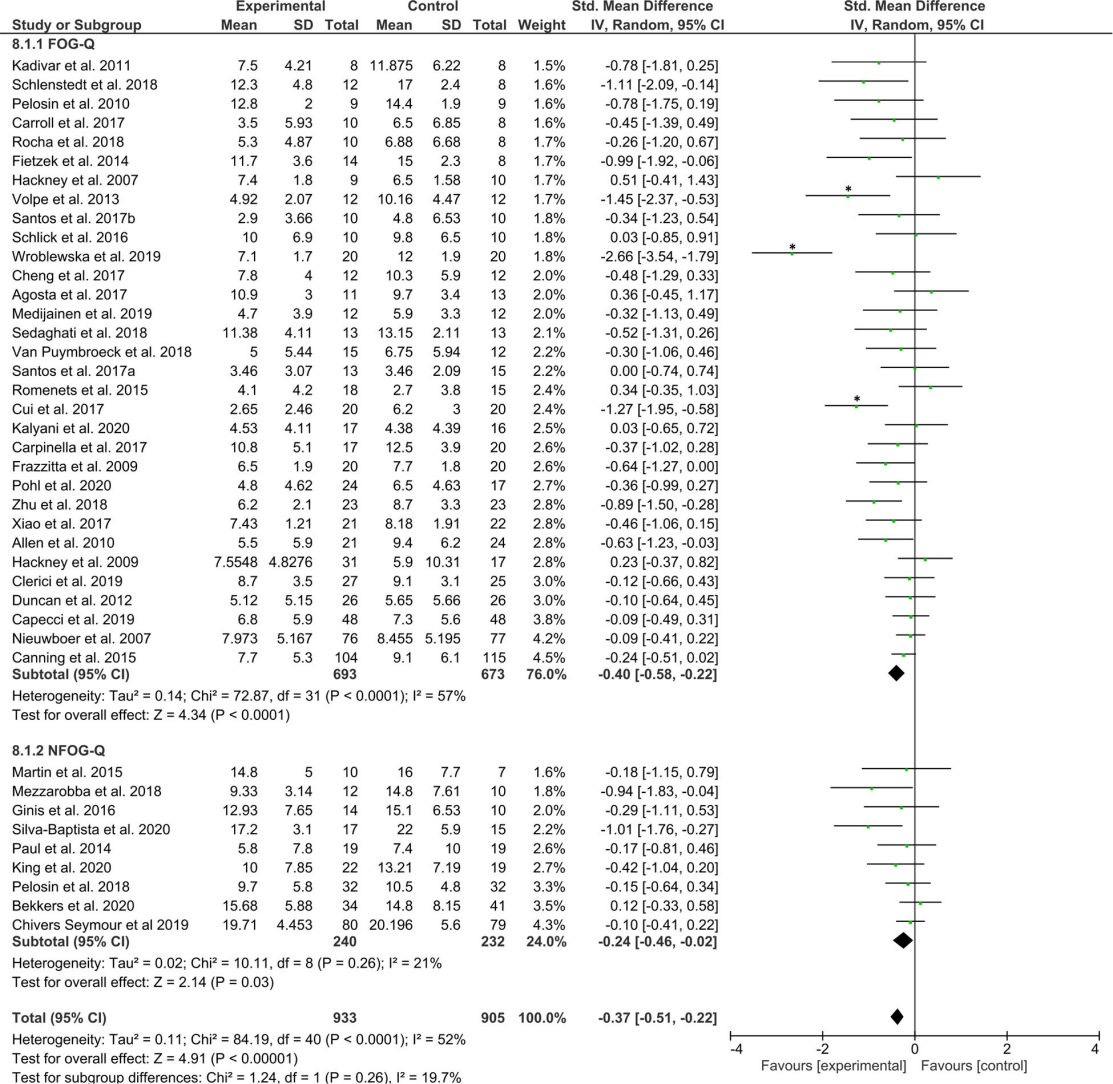
Primary analysis comparing all types of training/exercise interventions against any type of control group. The studies are arranged per weight of the study determined by sample size; *significant outliers removed in the sensitivity analysis.

The primary objective was to assess the effects of any exercise or training-based intervention on FOG in PD as compared to a control intervention. The primary meta-analysis was therefore conducted across all studies reporting either the FOG-Q or NFOG-Q as an outcome (*n* = 41 studies, totaling *N* = 933 and *N* = 905 subjects in the intervention and control groups, respectively). The test for overall effect revealed a favorable moderate effect for exercise/training compared to any type of control group (*Z* = 4.91, *p* < 0.00001), effect size (ES, [95% confidence intervals] = −0.37 [−0.51, −0.22]), but with large statistical heterogeneity across study effects (*χ*^2^(40) = 84.19, *p* < 0.01 and *I*^2^ = 52%). The forest plot of the primary analysis is presented in Fig. 3. The sensitivity analysis revealed that the heterogeneity assessed with the *χ*^2^ statistic became non-significant (*p* > 0.05) after removal of the outlier studies: Wroblewska et al.^54^, Volpe et al.^72^, and Cui et al.^87^. After excluding these three studies, 38 studies (*N*_active_ = 881, *N*_control_ = 853) remained, which revealed that the overall effect was still significant (*Z* = 4.60, *p* < 0.00001, ES = −0.24 [−0.35, −0.14]), but now with little statistical heterogeneity across study effects (*χ*^2^(37) = 40.48, *p* = 0.32 and *I*^2^ = 9%). Funnel plots are presented in Supplementary Data 1.

Available data of two studies could not be entered into the meta-analysis. Walton et al. conducted a cognitive training intervention designed to target the underlying correlates of FOG (i.e., attention and executive functioning) and compared its effects to that of a control cognitive training aimed at memory functioning, which is considered unrelated to FOG. Importantly, this was the only RCT to capture the percentage of time spent with FOG (%FOG) from video footage of a standardized gait assessment as the primary outcome^50,88^. Cognitive training targeting FOG reduced FOG severity during the ON medication state in those who experienced FOG during the baseline assessment (*N*_intervention_ = 20, *N*_control_ = 18, *p* = 0.002)^50^. This indicates that cognitive training may help to increase the processing capacity of the compensatory circuits involved in PD gait control, thereby reducing the amount of FOG (Fig. 1). No such improvement was seen during the OFF medication state (*p* = 0.80)^50^. King et al. compared the effects of different delivery modes of the same physical therapy intervention (i.e., Agility Boot Camp exercise program). Delivery was provided via an unsupervised home-based program (*n* = 17) for 4 weeks, or by individual- (*n* = 21) or group-class programs (*n* = 20), supervised 3× per week for 4 weeks. Their investigations revealed that group-based supervised delivery successfully reduced subjective FOG (*p* < 0.01), whereas both individually supervised (*p* = 0.431) and unsupervised home-based delivery (*p* = 0.308) did not. Not all patients had FOG, but the number of freezers in each group was roughly equivalent (46% for individual- and group-classes vs. 60% in the home-based group)^79^.

### Secondary analyses outcomes

**Fig. 4:**
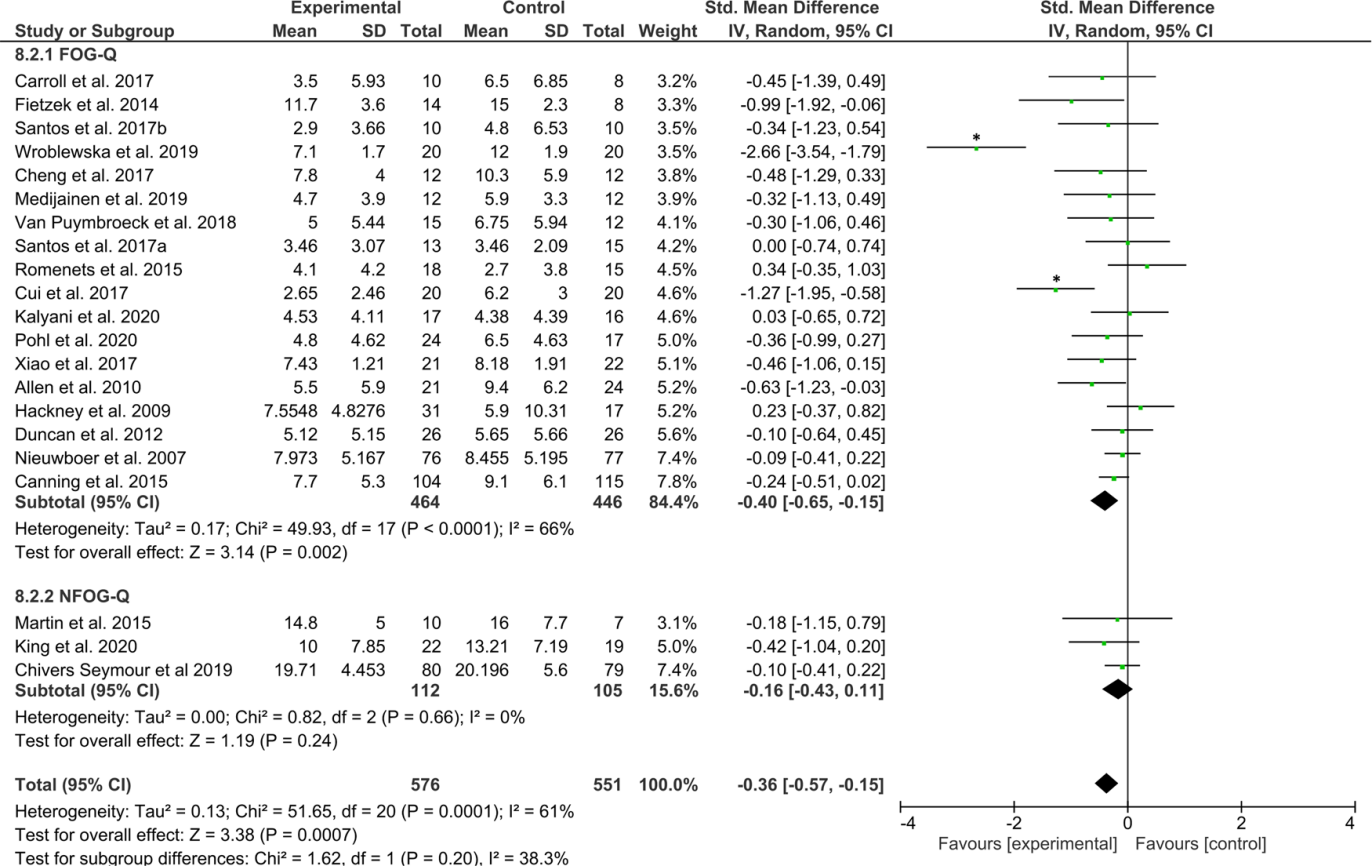
Secondary analysis comparing the effect of all types of training/exercise interventions against passive control groups. The studies are arranged per weight of the study as based on sample size; *significant outliers removed in the sensitivity analysis.

Secondary analyses were conducted to explore the main findings. Firstly, to indicate that training/exercise interventions are superior in reducing FOG severity to no intervention, a meta-analysis was conducted on 21 studies that compared the effects of training/exercise (*N* = 576) against a passive (i.e., wait-list, delayed-start, usual care) control group (*N* = 551) whom received no intervention. The test for overall effect revealed a significant moderate effect favoring the interventions (*Z* = 3.38, *p* = 0.0007, ES = −0.36 [−0.57, −0.15]), but again with high levels of statistical heterogeneity across study effects (*χ*^2^(20) = 51.65, *p* < 0.001, *I*^2^ = 61%). The Forest plot is presented in Fig. 4. A sensitivity analysis revealed that the statistical heterogeneity dropped to (*χ*^2^(18) = 13.3, *p* = 0.77, *I*^2^ = 0%) after excluding the studies by Wroblewska et al.^54^ and Cui et al.^87^, while the test for overall effects remained significant (19 studies, *N*_intervention_ = 536, *N*_control_ = 511, *Z* = 3.18, *p* = 0.001, ES = −0.20 [−0.32, −0.08]). Funnel plots for each of the secondary analyses are presented in Supplementary Data 1.

**Fig. 5:**
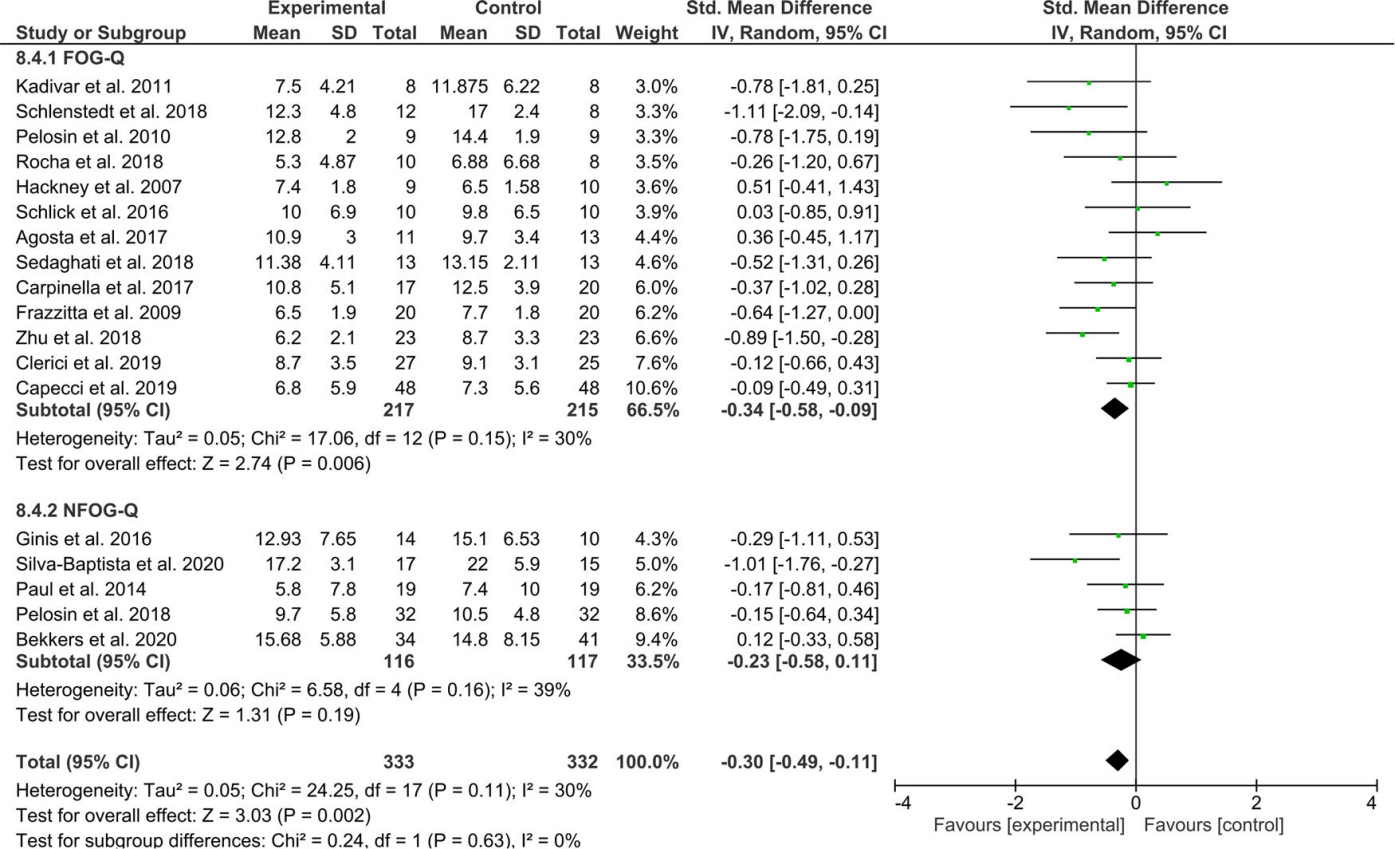
Secondary analysis comparing the effect of all types of training/exercise against active control groups. The studies are arranged per weight of the study as based on sample size.

A total of 18 studies compared the intervention of interest to an active control of similar exposure. Synthesizing their combined effects across *N*_intervention_ = 333 and *N*_control_ = 332 revealed a favorable moderate effect for the intervention groups (*Z* = 3.03, *p* = 0.002, ES = −0.30 [−0.49, −0.11]) with acceptable statistical heterogeneity (*χ*^2^(17) = 24.25, *p* = 0.11, *I*^2^ = 30%) and no significant outliers. The forest plot is presented in Fig. 5.

**Fig. 6:**
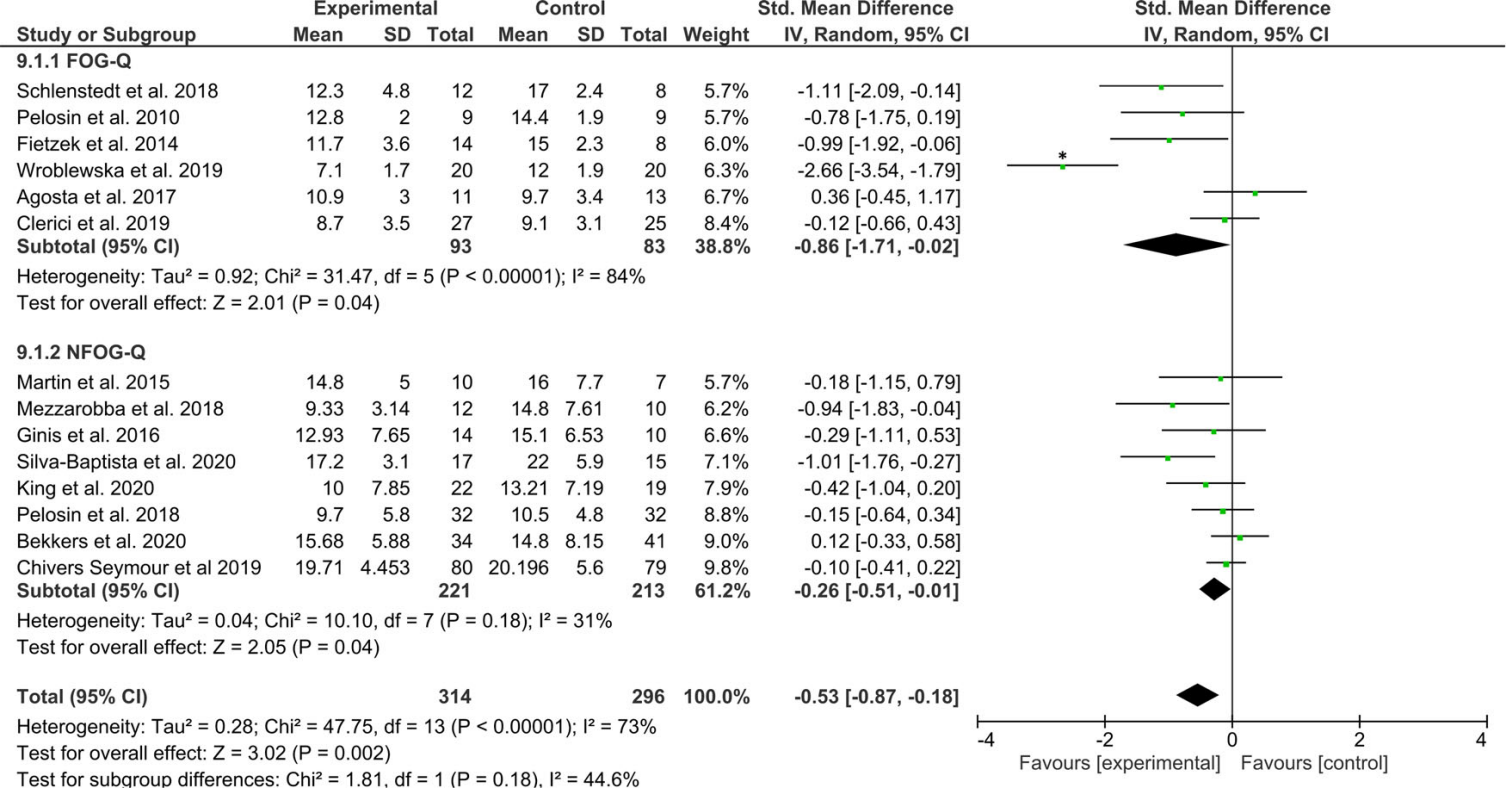
Secondary analysis comparing all types of training/exercise interventions against any type of control group in studies enrolling the only PD with FOG (i.e., freezers). The studies are arranged per weight of the study as based on sample size; *significant outliers removed in the sensitivity analysis.

Not all studies included only patients with FOG. As such, a secondary analysis was performed on 14 studies that only enrolled PD patients who experienced FOG (*N*_intervention_ = 314 and *N*_control_ = 296). The test for overall effect showed a significant moderate to large effect favoring the interventions compared to any type of control group (*Z* = 3.02, *p* = 0.002, ES = −0.53 [−0.87, −0.18]), but with high statistical heterogeneity across study effects (*χ*^2^(13) = 47.75, *p* < 0.00001, *I*^2^ = 73%) (Fig. 6). A sensitivity analysis revealed that the heterogeneity in the overall effect was mainly driven by Wroblewska et al., exclusion of which resulted in (*χ*^2^(12) = 19.31, *p* = 0.08, *I*^2^ = 38%) and a significant moderate effect still favoring the intervention (*Z* = 2.71, *p* = 0.007, ES = −0.32 [−0.55, −0.09]). Of these studies, ten designed the intervention to specifically target FOG or its underlying correlates (category A and B)^40,41,43–46,48,49,53,55^. Wroblewska et al. also mention that the Nordic walking training was delivered specifically for FOG, though the authors provided no hypothesis on how the intervention would indeed reduce FOG^54^. The home-based smartphone delivered automated feedback training intervention trialed by Ginis et al. was aimed at improving gait in PD, including, but not specific to FOG^70^. Similarly, the virtual-reality treadmill training studied by Bekkers et al. signified a secondary analysis on FOG of a trial that was originally designed to reduce falls^89^. Chivers-Seymour et al. trialed the PD-SAFE program designed for fall prevention in PD and not for FOG in particular^82^. Exploratory removal of these three studies, as well as the significant outlier study of Wroblewska et al.^54^, revealed that the overall effects of the remaining studies specific to FOG (*N*_intervention_ = 166, *N*_control_ = 146) remained statistically significant (*Z* = 3.08, *p* = 0.002, ES = −0.46 [−0.76, −0.17]), with acceptable statistical heterogeneity across study effects (*χ*^2^(9) = 13.8, *p* = 0.13, *I*^2^ = 35%). Importantly, this indicates that training-based interventions specifically targeting FOG are effective in reducing subjective FOG severity in PD freezers.

For the second research question of this review, we categorized the interventions according to their relevance to FOG (Box 1). The interventions were split into those that specifically aimed to alleviate FOG episodes or circumvent FOG-provoking situations (12 studies in meta-analysis), those that targeted the underlying motor and/or non-motor correlates of FOG (13 studies); and finally, those that offered generic exercise or physical therapy for other health benefits, except FOG (16 studies).

**Fig. 7:**
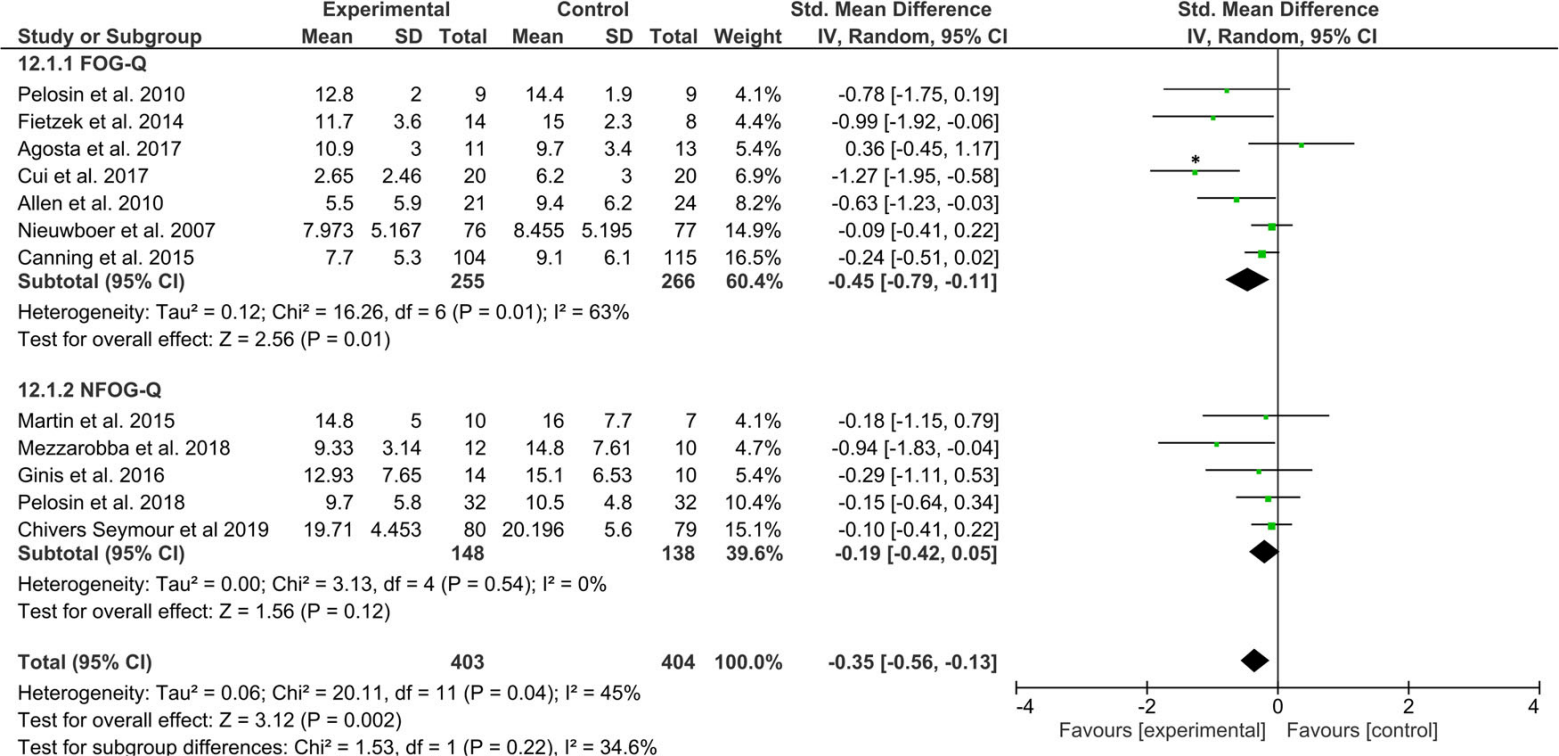
Secondary analysis comparing effects of category A interventions aimed directly at the alleviation of FOG episodes or FOG-provoking triggers against any type of control group. The studies are arranged per weight of the study as based on sample size; *significant outliers removed in the sensitivity analysis.

The first category of studies, which were at least partly directed at the alleviation of FOG episodes (*N*_intervention_ = 403 and *N*_control_ = 404 patients), revealed a significant moderate effect, favoring the interventions (*Z* = 3.12, *p* = 0.002, ES = −0.35 [−0.56, −0.13]) with some statistical heterogeneity across study effects (*χ*^2^(11) = 20.11, *p* < 0.04, *I*^2^ = 45%) (Fig. 7). A sensitivity analysis without an outlier^87^, revealed that the test for overall effects remained significant (*Z* = 2.84, *p* = 0.005, ES = −0.24 [−0.40, −0.07]) with acceptable levels of statistical heterogeneity across study effects (*χ*^2^(10) = 11.57, *p* = 0.31, *I*^2^ = 14%).

**Fig. 8:**
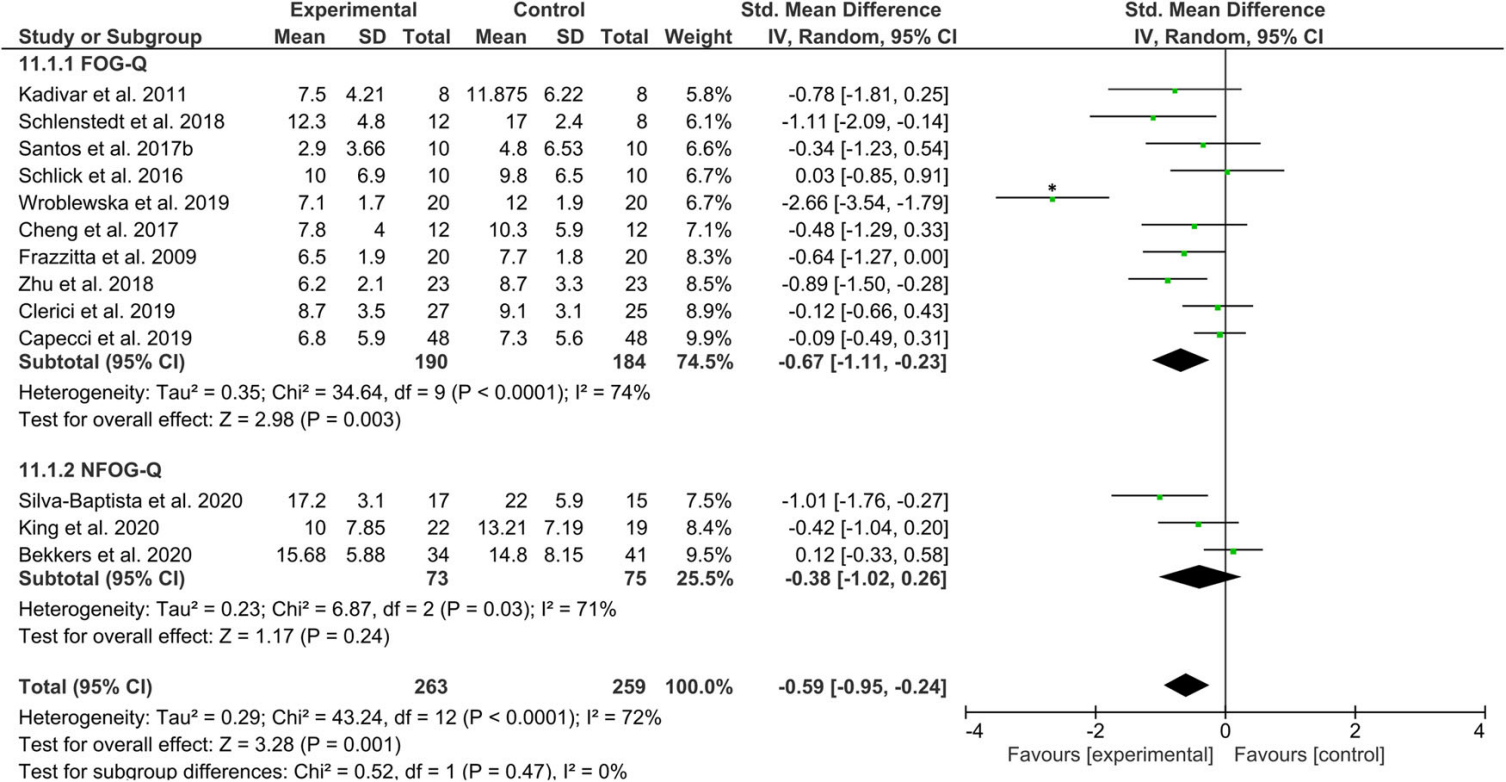
Secondary analysis comparing effects of category B interventions aimed at the underlying correlates of FOG against any type of control group. The studies are arranged per weight of the study as based on sample size; *significant outliers removed in the sensitivity analysis.

The second category, aimed at the underlying correlates of FOG, including 263 (*N*_intervention_) and 159 (*N*_control_) patients revealed a significant moderate effect favouring the intervention (*Z* = 3.28, *p* = 0.001, ES = −0.59 [−0.95, −0.24]), but again with high statistical heterogeneity across study effects (*χ*^2^(12) = 43.24, *p* < 0.0001, *I*^2^ = 72%), which was mainly driven by the study of Wroblewska et al.^54^ (Fig. 8). A sensitivity analysis without this outlier showed that the test for overall effects remained significant in favor of the intervention (*Z* = 3.27, *p* = 0.001, ES = −0.40 [−0.64, −0.16]) with acceptable levels of statistical heterogeneity across study effects (*χ*^2^(11) = 17.18, *p* = 0.10, *I*^2^ = 36%).

**Fig. 9:**
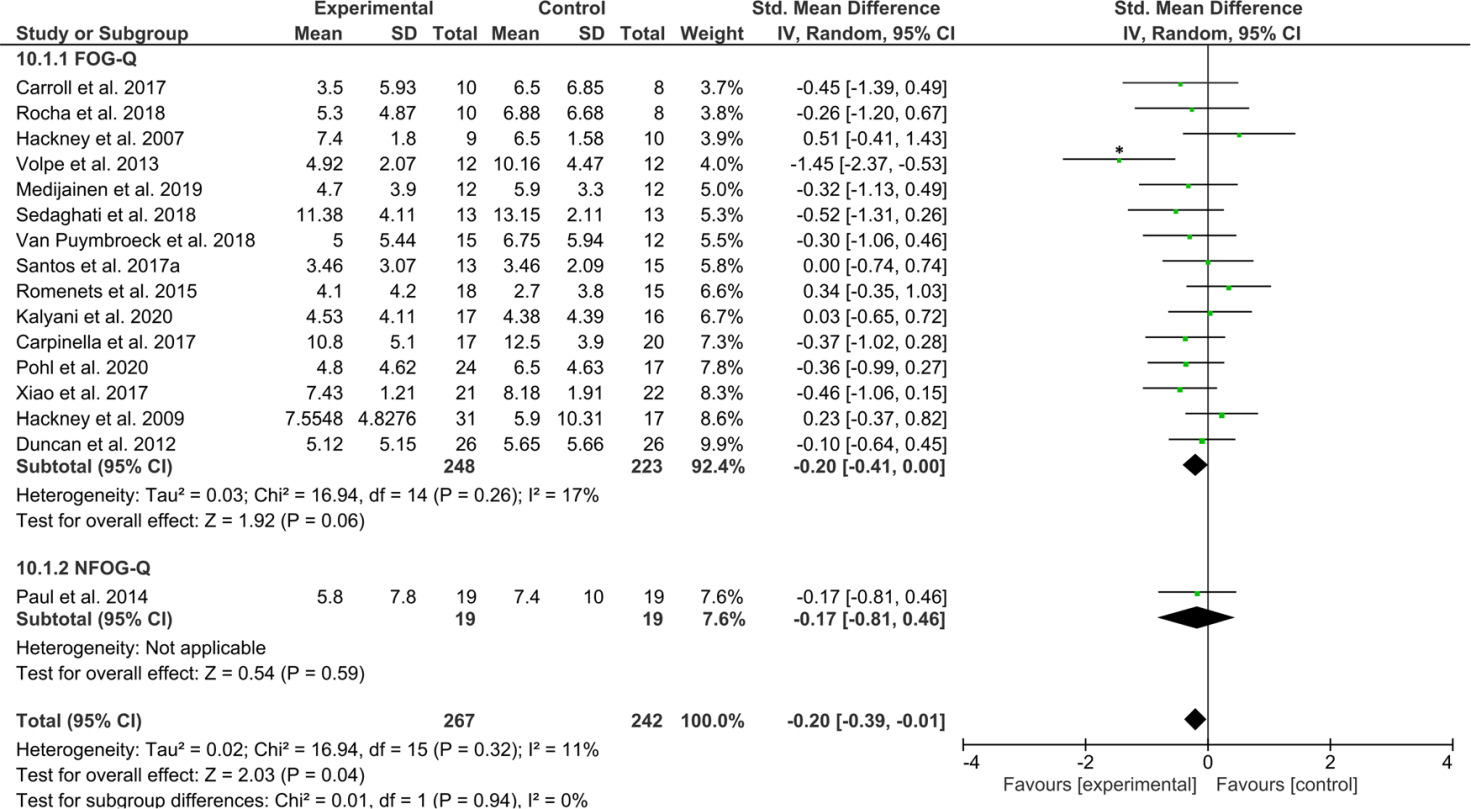
Secondary analysis comparing category C “generic exercise” interventions against any type of control group. The studies are arranged per weight of the study as based on sample size; *significant outliers removed in the sensitivity analysis.

The third and final subtype analysis on general exercise interventions across a total of *N*_intervention_ = 267 and *N*_control_ = 242 patients revealed a significant, yet small effect of exercise compared to any type of control group (*Z* = 2.03, *p* = 0.04, ES = −0.20 [−0.39, −0.01]), with little statistical heterogeneity across study effects (*χ*^2^(15) = 16.94, *p* = 0.32, *I*^2^ = 11%) (Fig. 9). The funnel plot, however, indicated that the effects were likely driven by a significant outlier^72^ (Supplementary Data 1). The overall small effect was indeed no longer significant after removal of this one study (*Z* = 1.57, *p* = 0.12, ES = −0.14 [−0.32, 0.04], *χ*^2^(14) = 9.42, *p* = 0.80, *I*^2^ = 0%).

**Fig. 10:**
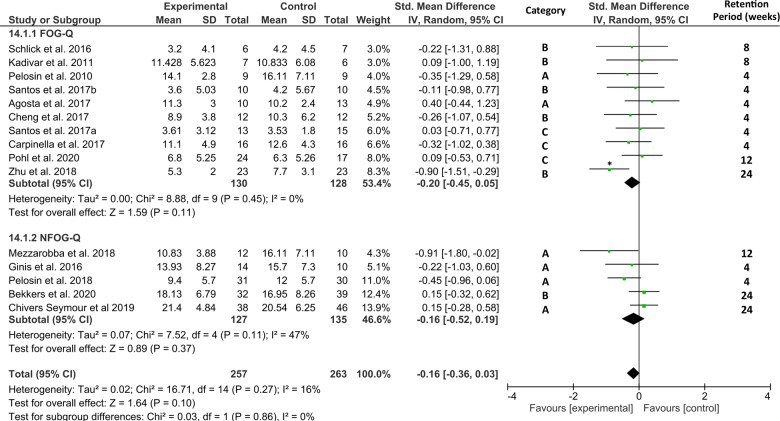
Secondary analysis comparing retention effects of all types of training/exercise interventions against any type of control group. The studies are arranged per weight of the study as based on sample size; *significant outliers removed in the sensitivity analysis.

To explore whether training/exercise interventions continue to reduce self-reported FOG beyond the intervention period, a final secondary retention analysis was performed on data from a subgroup of 15 studies that assessed the (N)FOG-Q at follow-up (1–6 months) after a period without training. Figure 10 shows no favorable effect of the interventions for reducing subjective FOG severity at follow-up (*N*_intervention_ = 257, *N*_control _= 263, *Z* = 1.64, *p* = 0.10, ES = −0.16 [−0.36, 0.03]) with little heterogeneity across study effects (*χ*^2^(14) = 16.71, *p* = 0.27, *I*^2^ = 16%). Inspection of the funnel-plot showed that one of these studies^51^ was a significant outlier, see Supplementary Data 1. Removal of this study lowered the statistical heterogeneity to (*χ*^2^(13) = 10.32, *p* = 0.67, *I*^2^ = 0%), while the overall small effect remained non-significant (*Z* = 0.91, *p* = 0.36, ES = −0.08 [−0.27, 0.10]). Moreover, three of the included studies^61,75,90^ were of the generic exercise category and thus not designed to specifically counter FOG. Removal of these three studies along with the one outlier^51^ made no difference to the overall effect size, which was still too small to reach statistical significance (*Z* = 0.88, *p* = 0.38, ES = −0.09 [−0.30, 0.11]) with little heterogeneity (*χ*^2^(10) = 9.48, *p* = 0.49, *I*^2^ = 0%).

## Discussion

This systematic review with meta-analysis assessed the published effects of exercise- and training-based interventions to reduce FOG-severity in PD. The primary meta-analysis, including 41 studies and 1838 PD patients, revealed a favorable and small-to-moderate effect of a wide variety of training modes for reducing subjective FOG-severity compared to any type of control condition, and this with acceptable levels of statistical heterogeneity across study effects. Moreover, exercise/training of any type proved more beneficial for reducing self-reported FOG severity than no intervention, or an active control condition. Importantly, training/exercise offered to only freezers, a notoriously more severely affected subgroup of PD on both motor and non-motor outcomes, proved also more beneficial than any type of control intervention, constituting the most salient finding of this study. However, no retention effects were found, indicating that the effects of exercise/training were not sustained beyond the intervention period. Finally, our subgroup analysis revealed that interventions aimed directly at reducing FOG and those aimed at the underlying correlates of FOG were both successful in reducing self-reported severity, whilst generic exercises were not.

The outcomes of the primary analysis assessing the (N)FOG-Q scores after any type of exercise/training compared to any type of control condition revealed a modestly positive and highly significant effect of the various exercise/training interventions for reducing subjective FOG in PD. These effects also remained significant and in favor of the exercise/training interventions when compared to either passive- or active control conditions. These findings thereby corroborate the outcomes of a recent meta-analysis^37^, whilst including data from additional studies increasing the robustness of these prior findings. More importantly, they indicate that diverse modes of exercise and/or training can be offered to PD to reduce the impact of FOG. Careful interpretation is warranted, however, given that many interventions were not directly aimed at reducing FOG, or FOG-related deficits, and included non-freezers. Item 1 of the NFOG-Q enables the exclusion of non-freezers, whom would all have scored zero on this outcome. The “original” FOG-Q, however, does not exclude non-freezers and contains two non-FOG-specific items. As such, non-freezers can score a maximum of 8 points on this scale^64^. Given that several studies included non-freezers, it cannot be established with certainty that the findings were truly FOG-specific. For this reason, we also conducted a meta-analysis in freezers only, underscoring that training-based interventions were indeed effective in this disease-burdened target population. Although some of these studies utilized the original FOG-Q containing two non-FOG-specific items, the outcome of this sub-analysis provides even stronger evidence of positive training effects on FOG in PD.

Another pertinent question we aimed to address was: which exercise/training intervention is best for FOG? This question remains difficult to answer, given the large variety of interventions trialed to date and because only a few studies directly compared the effects of different modes of exercise/training^55,62,67,68,72,79,80^. Until now, no consensus exists on a gold-standard intervention to which all other interventions can be compared, reflecting the current arbitrary clinical approach. It would therefore have been, and probably still is, more pressing to compare exercise/training against a passive or sham-control condition to test whether the intervention of interest is indeed effective for reducing FOG, before comparing it against another intervention.

Despite these drawbacks, we found that generic exercises do not contribute to the alleviation of FOG, in contrast to FOG-specific and FOG-relevant training. Surprisingly, FOG-relevant (category B) training was even more effective than FOG-specific (category A) training. This is unlikely explained by demographic or clinical differences. The average age (category A = 68.7; B = 69.9), disease duration (A = 9.4, B = 8.5 years), UDPRS-III (A = 28.4; B = 28.8), and (N)FOG-Q scores (A = 12.3; B = 11.4) at baseline did not appear to be different between these groups as based on available data from studies included in the subgroup meta-analysis (Supplementary Table 1). The surplus value of FOG-relevant exercise may be explained by the fact that such training plays into the notion that FOG-episodes are triggered by context-dependent dysfunctional neural information from various (compensatory) neural regions, which ultimately influences the locomotor network and causes transient gait disruptions^13,14,17,19^. As such, FOG-relevant exercise may increase the robustness of this compensatory reserve and prevent or postpone the actual emergence of FOG. In line with other work in neurorehabilitation for PD, training-related alterations are most likely to strengthen neuroplasticity through modulating compensatory circuits rather than changing the affected regions, such as the posterior putamen^91^. However, as the precise etiology of FOG remains elusive, i.e., whether it results from a temporary disruption (or overburdening) in the compensatory networks that allows for FOG to emerge, a dysfunction in the core systems that triggers the episodes, or a combination, it is probably most adequate at present to target both. This is supported by the present finding that both the FOG-specific (targeted triggers mostly) and FOG-relevant (targeted determinants mostly) type interventions seemed efficacious for reducing subjective FOG severity.

**Fig. 11:**
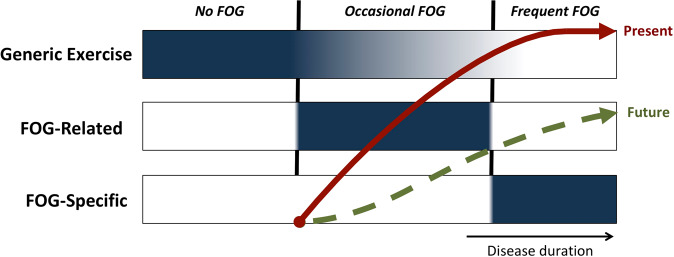
Theoretical framework for selecting the type of exercise/training in PD using a simple selection criteria, namely the self-reported frequency of FOG. Blue shaded areas indicate when this category of intervention is recommended. The red arrow represents the current progression of worsening FOG severity. The green dotted arrow represents the hypothesized attenuated progression of FOG severity that may be achieved by following this framework. Note that the intensity of generic exercise will likely need to be reduced when FOG-related interventions are offered due to time, energy, and resource constraints. There will also be an increasing need for supervised training by a therapist as the disease progresses and FOG becomes more regular.

What is more, our findings provide some new insights for translation to the clinical field. We propose a new framework for selecting the type of exercise/training for managing FOG using a very simple selection criterion, namely the frequency of FOG experienced (Fig. 11).

We suggest, together with others^92,93^, that conventional physical therapy and generic exercises should be made available to all PD patients. Generic exercise is best started early and maintained for as long as possible at a moderate to vigorous rate as this has been shown to enhance physical and mental fitness^92^, improve sleep quality^94^, and possibly even slow down motor symptom progression^93,95^. There is also indication that exercise increases striatal dopamine release in the anterior striatum^96^, and as such, exercise may help facilitate compensatory gait processing and subsequently delay the onset of FOG (Fig. 1), though this remains speculative at present. Early engagement in exercise may also lead to better adherence to long-term physical therapy interventions later in the disease^97^. Our review, as well as those of others^37^, have shown the benefit of a large variety of exercises (e.g., swimming, cycling, dancing, tai-chi, yoga, etc.) which can be offered according to the preferences (i.e., for optimal enjoyment) and abilities of each patient. Early in the disease when the risk of falling or other adverse events is relatively low, such generic exercises would not require constant supervision by trained staff, making it more widely accessible. Certain exercise programs, such as Tango dancing and Nordic walking, may also address some of the motor (e.g., gait, balance) and non-motor (e.g., cognitive) correlates of FOG and facilitate rhythmic movements via external cueing that could prove beneficial for pwPD who already experience FOG^54,77,98^.

Figure 11 illustrates that we further suggest that PD patients who present with occasional FOG (e.g., <1× week mostly in the dopaminergic OFF state), will likely benefit most from interventions targeting the underlying motor and non-motor correlates of FOG, making compensatory brain circuits more resilient against gait breakdown. Based on converging evidence, therapists may soon be able to predict which non-freezing patients have an increased risk for developing FOG in the future. Studies, so far, show that patients with mainly axial signs, gait difficulties, and repetitive movement breakdown are at risk for conversion to FOG^2,99^. These risk groups are likely to benefit from FOG-relevant interventions to reduce the impact of FOG in the future and perhaps even delay FOG onset. The most likely candidate training modes involve balance training^55,100^, turning practice^52^, cognitive training^50^, and combined motor-cognitive (i.e., dual-task) training^43,47,48^. Group-based classes could be considered whenever feasible for such interventions, as in one study these were shown to be most effective for reducing FOG^79^. The authors propose that the group-based classes may have challenged the patients more in terms of dividing attention and cognitive function than the individual delivery modes, which may thereby have resulted in these extra gains^79^. Indeed, recent studies showed that group programs combining such exercise also led to modest improvements of FOG^43,48^.

In the third column of our model (Fig. 11), we show that PD patients who are troubled by regular FOG are probably best-offered interventions aimed at directly managing FOG episodes. The most well-studied examples are cueing and cognitive rescue strategies to prevent or alleviate FOG^40,41,56,57^. Other training modes involve providing online gait feedback^70^, or preparing patients for FOG-provoking circumstances, such as through action-observation training^44–46,53^. Also, fall-prevention strategies in daily complex environments with the ultimate goal of helping patients cope with the FOG-related falls are part of this menu^74,82,86,101^. Given that anxiety can exacerbate FOG^11,102^, training behavioral strategies to better deal with the stress in anticipation of upcoming FOG, is also a viable FOG-specific intervention^103^, though in this systematic review we could not locate a study specifically trialing such a strategy. Therapists should closely supervise the delivery of FOG-related interventions to ensure correct performance, safety, and adjust difficulty levels over time for each patient^38^. To promote translation to everyday life, supervised home-based delivery may need to be considered^37,42^.

We speculate, as shown in Fig. 11, that it may be possible to shift the current projected evolution of FOG, as indicated by the red line, to a slower progression, as shown by the green dotted line. This possibility may be strengthened by capitalizing on the accumulative effect of FOG-relevant and FOG-specific interventions together. Moreover, offering a variety of FOG-related interventions might help keep patients motivated and their frontal attention circuits in an early motor learning state and thereby trained to better control motor operations. In further analogy to motor learning, a combination of aerobic exercise and motor training was found to have a synergistic effect, enhancing neuroplasticity in motor-related compensatory circuits in PD^104^. Careful selection of linking a specific mode of FOG-relevant training with a FOG-specific management approach may be also be favored to address the heterogeneity of FOG and its differing phenotypical manifestations^105^. It thereby remains imperative to keep improving our understanding of the determinants of FOG so that these can be targeted with future behavioral interventions and verify their impact experimentally.

However, the current study also revealed the lack of retention effects. Based on 15 studies, it became apparent that the effects of physical activity waned-off within 1–6 months after ceasing the intervention. This finding contradicts a recent meta-analysis, done on only eight studies, showing sustained retention effects after physical therapy^37^. The same eight studies were included in the present analysis, as were an additional seven. Also, the study of Zhu et al.^51^, which in our analysis was considered an outlier, was included in the study by Cosentino et al.^37^. Regardless, the lack of significant retention highlights the importance of keeping freezers engaged in exercise/training in the long-term, which is not self-evident in this population^38,97^. FOG itself, as well as prior falls and fear of falling, are in fact key predictors of poor adherence to exercise in PD^97^. Common non-motor symptoms, including anxiety, depression, cognitive decline, and pain are also predictive of poor adherence in PD^97^, highlighting the need for multidisciplinary care to achieve optimal disease management in freezers^106^. Freezers in particular may thus require extra motivation and follow-up. The outcomes of our review, fortunately, indicate that various training modes may be effective for FOG, offering a wide choice for patients. Adherence in the short-term at least was high (70–100%) for the many interventions reviewed here. This suggests that most people with PD are willing and able to engage in physical therapy interventions in the context of clinical research projects.

With the recent development of technology, another approach is to provide freezers with continuous intelligent cueing and feedback for gait. It is now increasingly possible to personalize cueing and make it fit directly to the motor output of the individual^107,108^. In addition, relatively low cost, lightweight, and unobtrusive Smartphone-based solutions will be leveraged in the near future to deliver cueing essentially at any time and in any setting. However, major impediments to the wide clinical use of wearable systems are that the development of valid algorithm to detect the freezing episodes online and accurately is still challenging.

There are several critical points that should be kept in mind when interpreting the outcomes of the present meta-analyses. First, as also previously noted by others^109^, the quality of reporting was fair at best for the majority of studies (Table 2) and did often not comply with the CONsolidated Standards of Reporting Trials (CONSORT) guidelines (consort-statement.org). Secondly, the dosage of the intervention varied a lot between the included studies. This difference was not taken into account in the present meta-analysis. Future studies could provide further insight into the association between treatment dosage and efficacy by means of a meta-regression. Thirdly, a different categorization of studies by intervention types would likely lead to different results in the subgroup analysis. Importantly, however, the categorization of studies in the present review was determined on an a priori basis and conducted by three independent assessors, thereby preventing confirmation bias. Of note is that one study on Nordic walking targeted FOG as the primary outcome^54^ and was therefore included in the “FOG-relevant” category, despite offering a conventional exercise type of intervention. Statistically, this study was considered an outlier, and its outcomes were thus not included in the sensitivity analyses. Lastly, only 11 studies defined FOG severity as the primary outcome^41,43–52^. Though, two of these studies investigated an intervention that was not specifically designed to target FOG^47,51^. A reason for not choosing FOG as a primary outcome may have been that FOG severity is notoriously difficult to assess given its unpredictable and episodic nature^1^. Patients with PD are prone to performance bias whereby they tend to switch to goal-directed gait control when being observed. This tendency has particularly negative effects for assessing FOG, because episodes occur less frequently in research or clinical settings^110^. Most studies to date, therefore, included subjective questionnaires to capture FOG severity, which are quick and easy to obtain. However, these scales can be biased by recollection errors, whereby patients may find it difficult to rate the severity of their symptoms in hindsight and scores may be influenced by a single troubling episode. Importantly, given that blinding of participants is difficult to achieve in exercise/training interventions, subjective ratings may be susceptible to placebo effects. Finally, our recent work has shown that the minimal detectable change of the NFOG-Q is 9.95 points, which constitutes about a third of the full range of scores^111^. Taken together, we do not recommend future studies rely solely on the (N)FOG-Q as an outcome of FOG severity.

Efforts are being made to develop responsive measures of FOG severity for use as outcomes in clinical trials, for instance by means of the semi-objective FOG score^69^ or by calculating the percentage of time spent frozen as rated from video-recordings of standardized FOG provoking walking tasks^50^. Although it is projected that the field will move towards fully objective at-home ratings of FOG severity as measured with inertial sensors, the limited accuracy of these systems to date for detecting FOG precludes their current use as a primary outcome^112^. We, therefore, recommend future phase-II and phase-III trials to be based on percentage time frozen obtained from video recordings of standardized walking tasks^50^ as the primary outcomes of choice for FOG severity^88^. Software to annotate the video recordings for FOG can be downloaded for free from morangilat.com^88^. The standardized protocol should consist of a substantial number of FOG-provoking tasks, such as turning on the spot^69,71,113^. The percentage time frozen can be rated by independent investigators who are kept blinded to group allocation^41,50,55^. Overall, the development of standardization procedures for assessment protocols and sensor-based methods is the precondition for further advancing trials that address novel therapeutic options for FOG.

## Conclusion

This systematic review with meta-analysis revealed a small-to-moderate effect size favoring various targeted training modes, but not generic exercise, for reducing the subjective impact of FOG in PD. Large-scale RCT’s investigating training interventions specifically aimed at reducing FOG using adequate outcomes are still urgently needed to further optimize the multidisciplinary management required for this common and disabling symptom in PD.

## Supplementary information

Supplementary Information

## Data Availability

The data that support the findings of this study are available within the paper and its Supplementary Information files.
