# Supplementary material for: A systematic review on exercise and training-based interventions for freezing of gait in Parkinson’s disease
Source: NPJ Parkinsons Dis. 2021 Sep 10;7:81. doi: 10.1038/s41531-021-00224-4 (PMC8433229; doi:10.1038/s41531-021-00224-4)

Supplementary Materials

Title: A systematic review on exercise and training-based interventions for freezing of gait in Parkinson's disease

Authors: Moran Gilat, Pieter Ginis, Demi Zoetewei, Joni De Vleeschhauwer, Femke Hulzinga, Nicholas D'Cruz, Alice Nieuwboer.

Included:
-Supplementary Note 1: PROSPERO registration
-Supplementary Table 1: Systematic overview of included studies
-Supplementary Data 1: Funnel Plots

Supplementary Note 1 - PROSPERO registration

Effect of rehabilitation on freezing of gait in Parkinson's disease: a systematic review and meta-analysis

Moran Gilat, Pieter Ginis, Nicholas D'Cruz, Alice Nieuwboer

Citation

Moran Gilat, Pieter Ginis, Nicholas D'Cruz, Alice Nieuwboer. Effect of rehabilitation on freezing of gait in Parkinson's disease: a systematic review and meta-analysis. PROSPERO 2019 CRD42019123882 Available from:
http://www.crd.york.ac.uk/PROSPERO/display_record.php?ID=CRD42019123882

Review question

The aim of this systematic review and meta-analysis is to summarize the effect of rehabilitation-based interventions on reducing the frequency and/or severity of freezing of gait (FOG) in people with Parkinson's disease (PD). We set out to answer the following questions:
i) Is rehabilitation effective in reducing Freezing of Gait (FOG) in Parkinson's disease (PD) compared to a control intervention or usual care?
ii) What is the nature and quality of the evidence?
iii) Can any recommendations be made for future intervention research based on these findings?

Searches

Based on recent guidelines by Bramer WM., et al. 2017*, we will search the following electronic databases: PubMed, MEDLINE Ovid, EMBASE, Web of Science (science citation index), and Google Scholar.
*https://doi.org/10.1186/s13643-017-0644-y

We will search for literature published online from inception of the databases without any date of language restrictions. The search will be re-run just prior to the final analysis and further eligible studies will be retrieved for inclusion. The specific inclusion and exclusion criteria are listed below.

Inclusion:
-Prospective empirical studies with a randomised controlled design with FOG in PD as either the primary or secondary outcome.
-Written in any language and without any date restrictions.

Exclusion:
-Not peer reviewed;
-Conference Abstracts;
-Reviews of the literature with- or without meta-analysis;
-Freezing only assessed at baseline;
-Only freezing during movements other than gait assessed (e.g. freezing during repetitive upper-limb movements or foot tapping).

The following search criteria will be applied (PubMed example):
"Parkinson disease"[MeSH] AND "Parkinson disease"[All fields] AND ("freezing"[All Fields] OR "festination"[all fields] OR "shuffling"[all fields]) AND ("gait"[all fields] OR "walking"[all fields]) AND (("rehabilitation"[All Fields] OR "rehabilitation"[MeSH Terms]) OR "Training"[all fields] OR "physiotherapy"[all fields] OR "physical therapy"[all fields] OR "non-pharmacological"[all fields] OR ("behavioural"[all fields] OR "behavioral"[all fields]) OR ("cueing"[all fields] OR "cues"[all fields]) OR "feedback"[all fields] OR ("action observation"[all fields] OR "action-observation"[all fields]) OR ("dual task"[all fields] OR "dual-task"[all fields]) OR "virtual reality"[all fields] OR ("exergame"[all fields] OR "exergaming"[all fields]) OR ("cognitive"[all fields]

OR "cognition"[all fields]) OR "auditory"[all fields] OR "visual"[all fields] OR "executive"[all fields] OR "sensory"[all fields] OR "proprioceptive"[all fields] OR "imagery"[all fields] OR "treadmill"[all fields] OR "wearables"[all fields] OR "dance"[all fields] OR "tango"[all fields] OR "tai chi"[all fields] OR "balance"[all fields] OR "strength"[all fields] OR "exercise"[all fields])

Types of study to be included

Inclusion:
-Prospective empirical studies with a randomised controlled design with FOG in PD as either the primary or secondary outcome.
-Written in any language without date restrictions.
Exclusion:
-Not peer reviewed;
-Conference Abstracts;
-Reviews of the literature with- or without meta-analysis;
-Freezing only assessed at baseline;
-Only freezing during movements other than gait assessed (e.g. freezing during repetitive upper-limb movements or foot tapping).

Condition or domain being studied

Freezing of Gait in Parkinson’s disease, Rehabilitation effects.

In comparison to two similar other registered reviews (PROSPERO CRD42018116820 and CRD42018086543) our review will focus solely on FOG-related outcome measures. These will only include measures directly related to FOG, such as freezing documented on video, freezing ratios measured with wearable sensors, freezing rated by a clinician, and self-reported freezing on diaries and freezing related questionnaires. In contrast to the other reviews, we will not assess proxy measures that are indirectly related to FOG, such as measures of gait, balance, falls, activities of daily living, and quality of life. Furthermore, we will: i) implement slightly different inclusion criteria (e.g. 2 days of training versus 2 weeks of training required), ii) Search for literature using a different combination of electronic databases; and iii) apply a different risk of bias assessment on the included studies.

Participants/population

Inclusion:

-Human participants with a diagnosis of idiopathic Parkinson’s disease
Exclusion:
-Human participants with a diagnosis of Parkinsonism or Parkinson’s Plus syndromes.
-Animal studies.

Intervention(s), exposure(s)

Inclusion:

-Any kind of training-based interventions of at least 2 days, such as behavioural therapy, physiotherapy, exercise programs, cueing interventions, action observation and dance therapy.

Exclusion:
-Pharmacological interventions
-Surgical or other invasive interventions
-Other non-rehabilitation based interventions, such as gene therapy.
-Non-invasive brain stimulation interventions, such as Transcranial Magnetic Stimulation (TMS) and transcranial Direct Current Stimulation (tDCS).

Comparator(s)/control
Active (i.e. Alternative intervention, Sham, Placebo) or Passive (i.e. Wait-list, Delayed start, No intervention besides usual care) control group.

Context
Freezing of gait (FOG) is one of the most debilitating symptoms of advancing Parkinson's disease (PD), leading to frequent falls and reduced quality of life. Conventional pharmacological and surgical treatments of PD are not adequately capable of alleviating FOG, which also becomes more and more difficult to treat as the disease inevitably progresses. Neurologists refer PD patients with FOG to rehabilitation, assuming that this will provide required relief. Rehabilitation is therefore emerging as an important adjunct therapy, whereby patients are trained behaviourally to manage their FOG and maintain daily functioning and independence for as long as possible. But, exactly how much scientific evidence is there for the overall beneficial effects of rehabilitation for FOG in PD, what is the quality of this evidence and what current training programs could we best offer patients to reduce their FOG severity? To our knowledge, no systematic review with meta-analysis has been performed to date to investigate these questions on FOG specifically.

Main outcome(s)

FOG is usually assessed subjectively using either the Freezing of Gait Questionnaire (FOG-Q), New-Freezing of Gait Questionnaire (NFOG-Q), Characterizing Freezing of Gait Questionnaire (C-FOGQ) or a FOG diary. FOG can also be assessed during clinical examination using specific items of the Movement Disorders Society Unified Parkinson's Disease Rating SCALE (MDS-UPDRS) Sections II and III, or objectively by calculating the number of FOG events or percentage of time spent Frozen from video recordings of an actual gait task, or by surrogate FOG measures obtained with wearable sensors, such as the FOG-ratio.

For the present review, any FOG outcome assessed as either the primary or secondary outcome of the intervention will be included for review. We expect that the majority of studies will have obtained the FOG-Q, given that this scale has been most widely applied in the research setting to assess FOG in people with PD. Pending the final number of studies included that have obtained the FOG-Q, we will conduct a meta-analysis on FOG-Q total scores. Given the anticipated heterogeneity of other FOG outcome measures obtained across studies, it remains to be determined whether any other FOG outcomes could be used for meta-analysis.

Additional outcome(s)
None

Data extraction (selection and coding)
Non-duplicate titles and abstracts extracted from the search strategy will be independently screened for eligibility by two reviewers (MG and PG). Any study that potentially meets the inclusion criteria will be identified, and their respective full texts screened for final eligibility. A third moderator (NC) will be consulted for discussion on any inconsistencies between the reviewers regarding the eligibility of studies. A senior researcher (AN) will approve the final list of studies included.
A standardized form will be used to extract the following data from included studies:
1. Source: Study ID, Authors, Study Title, Year of Primary Reference.
2. Methods: Study design, Study duration, Random sequence generation, Allocation sequence concealment, Blinding of participants and personnel, Blinding of outcome assessment; Selective reporting; Other sources of bias.
3. Participants: Total number of participants enrolled, Setting, Diagnostic criteria, Age, Sex, Disease severity, Cognition, Co-morbidity.
4. Interventions: Total number of intervention groups, Setting, Type, Dose, Details, Medication status during training (on/off)
5. Outcomes: Definition, Units of measurement; Time points collected, Time points reported.
6. Results: Total number of participants allocated to each intervention. For each outcome of interest: Sample size analysed at each time point; Missing participants at each time point; Summary data or effect estimates for each intervention group; Adverse events; Medication status during assessments (on/off).
7. Miscellaneous: Funding source; Key conclusion of the study authors; Miscellaneous comments by the

study authors; Miscellaneous comments by the review authors.
The two reviewers (MG, PG) will extract the 'Outcomes' and 'Results' data independently and any inconsistencies between entries that could not be resolved via discussion between the reviewers will be resolved by arbitration of a third moderator (NC). In the event of missing data, the corresponding study author will be contacted by email with a request to enter the missing summary items in a standardized data collection form that we will provide.

Risk of bias (quality) assessment

Two independent reviewers (MG and PG) will conduct the independent screening of titles, abstracts and full-texts, and disagreements will be resolved through discussion with a third moderator (NCD).
Individual study and reporting quality as well as susceptibility to bias will be assessed using the NIH National Heart, Lung and Blood Institute's Quality Assessment Tool for Controlled Intervention Studies. This tool uses 14 criteria for assessing internal validity and potential risk of bias.

Strategy for data synthesis

The objective is to assess the global effects of a rehabilitation-based intervention compared to a control intervention on FOG in PD. A table displaying the study characteristics and variations will be included. Where sufficient numbers of studies have obtained the same construct of FOG outcome measure, we will combine their outcomes using standardization when possible and pool the results using an inverse variance random-effects meta-analysis in RevMan (v5.3). We anticipate that a meta-analysis will be possible with the total FOG-Q and NFOG-Q as the main continuous outcome measures. The results will be described in text and table or Forest plot. For each outcome, standardized means for continuous outcomes and risk ratios for binary outcomes will be calculated with 95% confidence intervals and two sided P values. P-values <0.05 will be considered statistically significant. Heterogeneity between the studies in effect measures will be explored using both the $?^2$ and I^2 statistic. An I^2 value greater than 50% will be considered representative of substantial heterogeneity. Where high heterogeneity exists, sensitivity analyses will be conducted. Stratified meta-analyses may be employed to explore heterogeneity in effect estimates according to: study quality; study populations, and intervention content. We will also explore evidence of publication bias. If it is not possible to perform a meta-analysis, a narrative synthesis of the quantitative data will be provided using the Grading of Recommendation, Assessment, Development and Evaluation (GRADE) method (Murad HM., et al. 2017).
We intend to conduct the meta-analysis on data retrieved from studies with a low or unclear summary risk of bias. Depending on the number of studies with a high summary risk of bias, we will consider presenting stratified analysis for: i) only low risk studies; ii) all studies.

Analysis of subgroups or subsets

If sufficient data are available, exploratory analyses may be performed to assess:
-The effect of intervention type;
-The effect of intervention dosage;
-The effect of adherence to the intervention;
-The effect of the number of participants with FOG at baseline;
-The influence of disease severity on the effects of the intervention;
-The influence of dopaminergic medication status (on, off) during training and the assessment of FOG;
-The influence of cognition on the effects of the intervention.

Contact details for further information

Moran Gilat
moran.gilat@kuleuven.be

Organisational affiliation of the review

KU Leuven
https://www.kuleuven.be/english/

Review team members and their organisational affiliations

Dr Moran Gilat. KU Leuven
Dr Pieter Ginis. KU Leuven
Mr Nicholas D'Cruz. KU Leuven
Professor Alice Nieuwboer. KU Leuven

Type and method of review
Meta-analysis, Systematic review

Anticipated or actual start date
01 February 2019

Anticipated completion date
01 June 2019

Funding sources/sponsors
None

Conflicts of interest

Language
English

Country
Belgium

Stage of review
Review Ongoing

Subject index terms status
Subject indexing assigned by CRD

Subject index terms
Gait; Gait Disorders, Neurologic; Humans; Parkinson Disease

Date of registration in PROSPERO
22 March 2019

Date of publication of this version
22 March 2019

Details of any existing review of the same topic by the same authors

Stage of review at time of this submission

Stage	Started	Completed
Preliminary searches	Yes	No
Piloting of the study selection process	Yes	No
Formal screening of search results against eligibility criteria	No	No
Data extraction	No	No
Risk of bias (quality) assessment	No	No
Data analysis	No	No

Versions
22 March 2019

PROSPERO
This information has been provided by the named contact for this review. CRD has accepted this information in good faith and registered the review in PROSPERO. The registrant confirms that the information supplied for this submission is accurate and complete. CRD bears no responsibility or liability for the content of this registration record, any associated files or external websites.

Supplementary table 1: Systematic overview of included studies

Study (author, year)	Design	Outcome	FOG Obj.	Groups	Intervention	Dosage of intervention	N	% FOG	Age (years)	Sex (M, F)	DD	HY	UPDRS-III	LEDD	Assessment time points	Main findings regarding FOG
**Category A - FOG-Specific: Interventions aimed at reducing FOG episodes**																
Ashburn 2019[40] & Chivers Seymour 2019[41]**	Parallel groups	NFOG-Q	3	A	PDSAFE: a home-based fall prevention training program including strategies to overcome FOG	6 months, 12 supervised sessions at home, 60-90min + instruction to exercise 30min unsupervised each day	238	64	71 ± 7.7	147, 91	8 ± 6.6	2.58 ± 0.9	NR	NR	End of treatment (6 months) and 6 months follow-up	Outcomes on FOG not reported, but authors provided data for inclusion in the meta-analysis. No treatment effects found in a subgroup (A=80; C=79) of patients with FOG (NFOGQ item 1>0).
				C	Passive: usual care	NA	236	59	73 ± 7.7	119, 117	8 ± 5.8	2.7 ± 0.9	NR	NR		
Mezzarobba 2017[42]**	Parallel groups	NFOG-Q	1	A	AO + sonification of motor gestures designed to circumvent FOG	8w, 2pw, NR	12	100	74.7 ± 5.9	7, 5	10.8 ± 3.4	2.3 ± 0.5	32.9 ± 8.7	973 ± 253	End of treatment (8w) and 1 and 3 months follow-up	NFOGQ reduced significantly in the AO + Sonification group post-intervention and up to 3 months follow-up, whereas the sham group showed no improvements (group comparison p≤0.001).
				C	Sham: training the same motor gestures using cues but no AO + sonification	Same as A	10	100	72.0 ± 5.9	7, 3	9.4 ± 4.9	2.3 ± 0.7	33.2 ± 14	983 ± 380		
Pelosin 2018[43]	Parallel groups	NFOG-Q	1	A	AO of motor gestures designed to circumvent FOG	5w, 2pw, 45min	32	100	70.4 ± 4.5	16, 17	10.7 ± 3.9	2.4 ± 0.5	31.6 ± 6.1	435 ± 159	End of treatment (5w) and 4 weeks follow-up	NFOGQ reduced significantly post-intervention in both groups (p<0.001), but these effects remained significant only after AO at 4 weeks follow-up (p<0.001).
				C	Sham: training the same motor gestures without AO	Same as A	32	100	72.8 ± 3.1	15, 16	9.5 ± 4.2	2.6 ± 0.3	30.9 ± 7.2	383 ± 270		
Agosta 2017[44]	Parallel groups	FOG-Q;<br><br>UPDRS-II FOG item	2<br><br>2	A	AO of motor gestures designed to circumvent FOG	4w, 3pw, 60min	12	100	69.0 ± 8.0	10, 2	NR	2.3 ± 0.4	27.6 ± 9.7	897 ± 508	End of treatment (4w) and 4 weeks follow-up	Both groups reduced FOG severity post-intervention (A: p=0.02, C: p=0.05). The AO group showed a reduction after 4-weeks follow-up (p=0.06), but no significant Group x Time interaction effect was found (p=0.77).
				C	Sham: training the same motor gestures without AO	Same as A	13	100	64.0 ± 7.0	8, 5	NR	2.2 ± 0.3	23.5 ± 7.9	988 ± 345		
Cui 2017[45]	Parallel groups	FOG-Q	2	A	Physical therapy with cueing exercises aimed at	4w, 5pw, 30min	20	NR	60.3 ± 7.2	12, 8	6.20 ± 2.3	NR	27.5 ± 4.3	350 (212 - 476)	End of treatment (4w)	Physical therapy with cueing exercises reduced FOG, whereas

					reducing FOG											physical therapy without cueing exercises did not (group comparison: p=0.034).
				C	Sham: conventional physical therapy without cueing	Same as A	20	NR	60.5 ± 6.6	11, 9	6.05 ± 2.7	NR	27.6 ± 3.1	246 (211 - 524)		
Ginis 2016[46]	Parallel groups	NFOG-Q	3	A	CuPiD: corrective feedback on gait performance aimed at reducing FOG	6w, 1pw supervised at home and 2pw unsupervised at home, 30min	20	70	67.3 ± 8.1	17, 3	10.7 ± 5.4	2.3 ± 0.44	28.3 ± 14.8	NR	End of treatment (6w)	No treatment effects found.
				C	Sham: similar gait training without feedback	Same as A	18	55.6	66.1 ± 8.1	13, 5	11.7 ± 7.4	2.2 ± 0.39	33.8 ± 14.4	NR		
Canning 2015[47]	Parallel groups	FOG-Q	2	A	Fall prevention exercises including cueing strategies aimed at reducing FOG	24w, 3pw, 40-60min	115	46	71.4 ± 8.1	69, 46	7.5 ± 5.8	2.7 ± 0.5	25.8 ± 8.9	787 ± 486	End of treatment (24w)	No treatment effects found.
				C	Passive: usual care	NA	116	53	69.9 ± 9.3	66, 50	8.3 ± 6.0	2.7 ± 0.6	26.7 ± 10.1	807 ± 521		
Martin 2015[48]	Cross-over	NFOG-Q	2	A	Cueing training aimed at FOG	4w, 1.5pw, 30-60 min supervised, then 20 weeks unsupervised at home	10	100	72 ± 5.1	7, 5	9.0 ± 4.6	2.9 ± 0.5	NR	NR	End of treatment (6 months)	No treatment effects found.
				C	Passive: wait-list	NA	9	100	72 ± 5.8	6, 3	13 ± 8.2	2.6 ± 0.7	NR	NR		
Fietzek 2014[49]	Cross-over	FOG-Q;<br><br>FOG score	1<br><br>2	A	Cueing training aimed at FOG	2w, 3pw, 30min intervention, followed by 2w wait-list	14	100	69.8 ± 6.5	9, 5	12.1 ± 6.4	3 (2-3)	NR	664 ± 243	End of treatment (2w), corrected for carry-over effects.	Severity of FOG on both outcomes improved significantly after the intervention as compared to the control period (p<0.01).
				C	Passive: wait-list	2w wait-list, followed by same intervention as A	8	100	64.2 ± 5.9	7, 1	13.3 ± 3.6	3 (2-3)	NR	566 ± 195		
Allen 2010[50]	Parallel groups	FOG-Q	2	A	Fall prevention exercises including cueing strategies aimed at reducing FOG	24w, 3pw, 40-60min unsupervised at home + monthly supervised classes	24	NR	66 ± 10	13, 11	7 ± 5	NR	29 ± 10	NR	End of treatment (24w)	FOG significantly improved over time in the intervention group as compared to the control group (p=0.03).
				C	Passive: usual care	NA	24	NR	68 ± 7	13, 11	9 ± 6	NR	30 ± 15	NR		
Pelosin 2010[51]	Parallel groups	FOG-Q;<br><br>FOG Diary	1<br><br>1	A	AO of motor gestures designed to circumvent FOG	4w, 3pw, 60min	9	100	68.8 ± 4.1	NR	11.6 ± 4.9	2.1 ± 0.3	17.5 ± 4.6	NR	End of treatment (4w) and 1, 2, 3, 4 weeks follow-up	Both outcomes reduced post-intervention in both groups (p<0.001). The FOGQ reduced more after AO than Sham (p<0.05) and the effects
				C	Sham: training the same motor gestures without	Same as A	9	100	70.2 ± 6.8	NR	9.5 ± 3.7	2.2 ± 0.3	20.6 ± 5.7	NR		

					AO											of AO on the FOG diary remained for 4 weeks (p<0.001), whereas sham only led to effects up to 1-week follow-up (p<0.001).
Nieuwboer 2007[52]**	Cross-over	FOG-Q	2	A	Cueing in the home aimed at FOG	3w, 3pw, 30min intervention, followed by 3w wait-list	76	20.3	67.5 (61.5-72)	48, 28	7 (4-11)	2.5 (2.5-3)	31 (25-37)	500 (300-700)	End of treatment (3w), corrected for carry-over effects.	No overall treatment effect, although FOG reduced by 5.5% after cueing in the subgroup of freezers only (p=0.007).
				C	Passive: wait-list	3w wait-list, followed by same intervention as A	77	20.9	69 (62.5-73)	40, 37	8 (4-12)	3 (2.5-3)	34 (28-41)	350 (200-500)		
**Category B - FOG-relevant: Interventions targeting the underlying correlates of FOG**																
Bekkers 2020[53]**	Parallel groups	NFOG-Q	2	A	Treadmill training with virtual reality	6w, 3pw, 45min	34	100	70.41 ± 6.1	20, 14	9.59 ± 5.7	2.60 ± 0.44	33.35 ± 14.7	NR	End of treatment (6w) and 6 months follow-up	No treatment effects found.
				C	Sham: Treadmill training without virtual reality	Same as A	43	100	70.70 ± 6.0	29, 14	11.1 ± 9.6	2.57 ± 0.47	30.63 ± 11.9	NR		
King 2020[54]	Cross-over	NFOG-Q;<br><br>FOG-ratio	1	A	Agility boot camp-cognitive (ABC-C) program	6w, 3pw, 80min	23	100	68.2 ± 5.4	NR	7.5 ± 4.3	2.2 ± 0.5	43.1 ± 10.2	NR	End of treatment (6w)	NFOGQ worsened in the education-first group and improved in the exercise-first group (effect size=0.42), while after the cross-over both groups scored slightly worse on the NFOGQ indicative of a possible regression towards the mean effect. The FOG-ratio decreased in the education-first group and remained unchanged in the exercise-first group, though the groups were not matched at baseline.
				C	Passive: education control group plus instructions for relaxation exercises at home	6w, 1pw, 80min + relaxation exercises totaling 240min/pw similar to A.	19	100	68.5 ± 9.1	NR	9.9 ± 6.2	2.5 ± 0.8	50.3 ± 15.1	NR		
Silva-Batista 2020[55]	Parallel groups	NFOG-Q;<br><br>FOG-ratio	1	A	Adapted resistance with instability motor-cognitive balance training	12w, 3pw, 80-90min	17	100	64.6 ± 10.5	12, 5	7.7 ± 4.0	3.1 ± 0.3	NR	437± 212	End of treatment (12w)	NFOGQ reduced after the intervention (p<0.0001), but not control (p=0.989), though the interaction was non-significant (p=0.069). The FOG-ratio worsened in the
				C	Sham: traditional motor rehabilitation	Same as A	15	100	66.8 ± 8.9	6, 9	10.0 ± 5.6	3.2 ± 0.4	NR	503 ± 186		

																control group (p=0.002) and trended towards improvement in the intervention group (p=0.078), resulting in a significant interaction effect (p<0.001).
Capecci 2019[56]	Parallel groups	FOG-Q;<br><br>UPDRS-II FOG item used to define freezers	2<br><br>2	A	Robot-assisted gait training	4w, 5pw, 45min	48	68	68.1 ± 9.8	19, 29	8.9 ± 5.3	3.0 ± 1.0	22.4 ± 9.5	740 ± 328	End of treatment (4w)	The FOGQ improved in both groups with equal %freezers, but more so after the intervention (3.1 points) compared to control (1.5 points) with a significant time x treatment effect (p=0.03). The same interaction effect was also seen in a subgroup analysis on freezers only (p=0.04).
				C	Sham: treadmill training	Same as A	48	63	67.0 ± 7.6	24, 24	8.9 ± 4.3	3.0 ± 1.0	24.9 ± 16.7	739 ± 301		
Clerici 2019[57]	Parallel groups	FOG-Q	1	A	Motor-cognitive training + aquatic training	4w, 5pw (of which 3pw were aquatic training), 60min	27	100	67 ± 8	19, 8	NR	2.7 ± 0.4	21.0 ± 3.3	919 ± 407	End of treatment (4w)	FOG improved over time in both groups (p<0.001), without a group x time interaction effect (p=0.58).
				C	Sham: motor cognitive training only	4w, 5pw, 60min	25	100	67 ± 11	20, 5	NR	2.7 ± 0.4	20.6 ± 6.8	951 ± 328		
Wróblewska 2019[58]	Parallel groups	FOG-Q;<br><br>FOG score	1	A	Nordic walking training	12w, 2pw, 60min	20	100	72.1 ± 7.5	12, 8	5.2 ± 1.1	2.55 ± 0.51	32.7 ± 6.9	NR	End of treatment (12w), and 3-month follow-up for Active group only	FOGQ decreased after the intervention (6.7 points) and deteriorated (1.4 points) in the control group, leading to significant group x time interaction (p<0.0001). However, FOGQ scores at baseline were much higher in the intervention (13.8 points) compared to control (7.9 points) group. The significant treatment effect might thus reflect regression to the mean.
				C	Passive: usual care	NA	20	100	67.6 ± 6.6	9, 11	6.0 ± 1.2	2.45 ± 0.51	32.0 ± 7.7	NR		
Schlenstedt 2018[59]	Parallel groups	FOG-Q;	1	A1	Resistance training	7w, 2pw, 60min	12	100	78.3 ± 58	9, 3	11.2 ± 6.6	2.8 ± 0.3	24.3 ± 10.0	765 ± 448	End of treatment (7w)	No treatment effects found.

		FOG score	1	A2	Balance training	Same as A1	8	100	81.4 ± 7.3	6, 2	8.4 ± 7.3	2.9 ± 0.5	25.8 ± 5.7	652 ± 286		
Walton 2018[60]	Parallel groups	%FOG from video	1	A	Cognitive training specific to FOG, aimed at attention and executive functioning	7w, 2pw, 120min	20	100	69.7 ± 7.6	14, 6	9.95 ± 4.4	2.78 ± 0.66	38.7 ± 13.7	829 ± 315	End of treatment (7w)	Cognitive training significantly improved FOG compared to sham during the ON-state in PD who experienced FOG at baseline (p=0.002). No effects were found during the OFF-state (p=0.80).
				C	Sham: cognitive training not specific to FOG	Same as A	18	100	69.6 ± 7.8	11, 7	5.30 ± 1.8	2.53 ± 0.65	33.4 ± 12.6	975 ± 571		
Zhu 2018[61]	Parallel groups	FOG-Q	1	A	Aquatic obstacle training	6w, 5pw, 30min	23	NR	65 (6)	NR	6.8 ± 2.6	2.37 ± 0.43	51.6 ± 18.4	NR	End of treatment (6w) and 6 months follow-up	Both forms of aquatic training significantly improved FOGQ (p<0.001), but more so in the aquatic obstacle training group compared to the regular aquatic training group (p=0.004).
				C	Sham: aquatic training without obstacles	Same as A1	23	NR	67 (5)	NR	6.7 ± 2.4	2.43 ± 0.41	50.9 ± 15.6	NR		
Cheng 2017[62]	Parallel groups	FOG-Q	1	A	Curved walking training	12 sessions over 4-6 weeks, 30 min	12	NR	65.8 ± 11.5	9, 3	6.1 ± 4.1	NR	19.7 ± 4.2	402 ± 199	End of treatment (4-6w) and 1 month follow-up	Curved walking training significantly reduced FOGQ post-intervention and at 1-month follow-up compared to control exercises (p=0.016).
				C	Sham: trunk and upper limb exercises	Same as A	12	NR	67.3 ± 6.4	8, 4	8.1 ± 4.6	NR	19.5 ± 6.3	421 ± 243		
Santos 2017b[63]	Parallel groups	FOG-Q	2	A	Slack line training	6w, 2pw, 23min	11	NR	73.1 ± 9.8	6, 5	10.7 ± 4.1	2.18 ± 0.60	9.72 ± 4.88	325 ± 235	End of treatment (6w) and 1 month follow-up	A mean reduction of 1.0 point on the FOGQ was found after slack line training (p<0.05), while the control group increased by 0.4 points. These effects did not remain after 4 weeks follow-up.
				C	NR	NR	11	NR	78.1 ± 5.2	5, 6	10.9 ± 3.2	1.9 ± 0.53	9.18 ± 8.01	326 ± 149		
Schlick 2016[64]	Parallel groups	FOG-Q	2	A	Treadmill training with visual cueing not targeted at FOG episodes	5w, 2-3pw, 12 sessions in total, 20-45min	10	NR	71.2 ± 11	2, 8	10.4 ± 5.2	2.8 ± 0.9	28.9 ± 13.8	NR	End of treatment (5w) and 2 months follow-up	No treatment effects found.
				C	Sham: treadmill training without visual cueing	Same as A	10	NR	68.9 ± 6.8	4, 6	9.1 ± 3.1	2.7 ± 0.7	25.3 ± 15.1	NR		
King 2015[65]	Parallel groups	FOG-Q	3	A1	Individually supervised agility boot camp training	4w, 3pw, 60min	21	46	64.2 ± 6.7	17, 4	7.9 ± 7.9	2.4 ± 0.5	39.4 ± 11.1	NR	End of treatment (4w)	An overall effect of intervention on FOGQ was found across the three groups (p=0.038). The group class showed
				A2	Supervised group-	Same as A1	20	46	63.9 ± 8.5	14, 6	5.4 ± 3.6	2.4 ± 0.5	35.4 ± 14.1	NR		

					class agility boot camp training											strongest improvement (-1.2 points, p=0.001), while no change was seen in the individual (-0.62 points, p=0.31) or home-based (+0.35 points, p=0.41) groups.
				A3	Unsupervised home-based agility boot camp training	Same as A1 (85% compliance)	17	60	64.6 ± 6.8	10, 7	5.2 ± 5.8	2.5 ± 0.5	35.2 ± 13.7	NR		
Ricciardi 2015[66]*	Parallel groups	FOG-Q	2	A1	Physiotherapy for the most affected body side	12w, 2pw, 60min	9	NR	66.0 ± 6.1	6, 3	10.8 ± 4.9	2.2 ± 0.6	26 ± 12.8	827 ± 217	1 and 3 months after starting the treatment and 1 month follow-up	FOG is listed as an outcome, but no data or results on FOGQ reported.
				A2	Physiotherapy for the least affected body side	Same as A1	9	NR	69.0 ± 5.8	6, 3	9.1 ± 4.6	2.7 ± 0.7	33.3 ± 12	725 ± 299		
				A3	Physiotherapy for both body sides	Same as A1	10	NR	70.0 ± 4.9	7, 3	12.6 ± 7.7	2.3 ± 0.7	24.8 ± 6.1	837 ± 396		
Kadivar 2011[67]**	Parallel groups	FOG-Q	2	A	Auditory cued stepping in place	6w, 3pw, 45-60min	8	50	73.3 (2.2)#	5, 3	8.9 (1.8)#	2.69 (0.6)#	27.1 (4.1)#	456 ± 318	End of treatment (6w) and 1, 4, 8 weeks follow-up	FOG significantly improved after rhythmic auditory cueing training compared to internally paced training and the effects remained up to 4 weeks follow-up (p<0.05).
				C	Sham: internally paced stepping in place	Same as A1	8	37.5	70.5 (2.2)#	6, 2	7.5 (1.2)#	2.69 (0.6)#	27.0 (3.8)#	509 ± 297		
Frazzitta 2009[68]	Parallel groups	FOG-Q	1	A	Treadmill and cueing training	4w, 7pw, 20min	20	100	71 ± 8	8, 12	13.2 ± 4.1	3 (all subjects)	21.6 ± 5.6	685 ± 246	End of treatment (4w)	FOG improved significantly in both groups (p<0.001), but more so in the treadmill and cueing training group (p=0.007).
				C	Sham: cueing training without treadmill	Same as A1	20	100	71 ± 7	9, 11	12.9 ± 4.6	3 (all subjects)	23.6 ± 5.2	720 ± 232		
**Category C - Generic Exercise: Interventions consisting of generic exercises or physical therapy not related to FOG**																
Kalyani 2020[69]	Parallel groups	FOG-Q	3	A	Dancing, both seated and standing	12w, 2pw, 60min	17	NR	65.2 ± 11.9	3, 14	3.76 ± 2.9	1.65 ± 0.79	38.71 ± 17.7	502 ± 332	End of treatment (12w)	Difference scores indicated that the FOGQ decreased more (-0.61 points) in the dance group than the control group (+1.3 points, p=0.02).
				C	Passive: usual care	NA	16	NR	66.5 ± 7.7	10, 6	5.94 ± 3.6	1.56 ± 0.81	30.25 ± 15.6	716 ± 418		
Pohl 2020[70]	Parallel groups	FOG-Q	3	A	Group- and music based Ronnie Gardiner method	12w, 2pw, 60min	26	NR	69.7 ± 7.0	19, 7	6.0 ± 4.4	2.5 ± 1.0	34.0 ± 12.9	728 ± 327	End of treatment (12w), and 3 months follow-up	No treatment effects found.
				C	Passive: usual care	NA	20	NR	70.4 ± 6.0	13, 7	6.8 ± 3.6	2.0 ± 1.0	28.6 ± 10.4	565 ± 328		
Hubble 2019[71]	Parallel groups	FOG-Q	2	A	Trunk exercises + fall education brochures	12w, 1pw, NR	11	NR	63.3 ± 4.9	7, 4	6.5 ± 5.2	1.8 ± 0.6	17.3 ± 14.4	565 ± 378	End of treatment (12w), and 3 months follow-up	Authors report that neither intervention led to a change in the FOGQ at the end of treatment, nor follow-up, though
				C	Sham: fall education	NA	11	NR	67.5 ± 5.8	8, 3	7.0 ± 5.0	2.0 ± 0.7	21.5 ± 11.7	868 ± 476		

					brochures only											the precise outcomes are not reported.
Medijainen 2019[72]	Parallel groups	FOG-Q	2	A	Physiotherapy for PD, including cueing but not specific to FOG	8w, 2pw, 60min	12	NR	71.1 ± 4.2	5, 7	8.0 ± 6.9	2.2 ± 0.5	39.1 ± 14.7	NR	End of treatment (8w)	FOGQ changed by -2.8 points on average in the intervention group (p=0.043), and 0.1points in the control group (p=0.89), but no between-group difference was found (p>0.05).
				C	Passive: wait-list	NA	12	NR	69.9 ± 5.1	5, 7	7.7 ± 5.4	2.3 ± 0.7	36.4 ± 18.4	NR		
Rocha 2018[73]	Parallel groups	FOG-Q	2	A1	Tango	8w, 1pw supervised + 1pw unsupervised at home, 60min	10	NR	70.2 ± 5.5	4, 6	7.2 ± 4.9	2.5 (2-4)[$]	NR	NR	End of treatment (8w)	No group effects found. An effect of time was found in the mixed-genre dance group (p=0.046, ES=0.37), but not in the Tango group.
				A2	Mixed-genre dance	Same as A1	11	NR	72.9 ± 5.5	4, 7	8.4 ± 5.2	2.5 (1.5-4)[$]	NR	NR		
Sedaghati 2018[74]	Parallel groups	FOG-Q	2	A	Alexander based corrective techniques on forward flexed posture	8w, 3xpw, 60min	13	NR	64.9 ± 2.6	8, 5	5.3 ± 1.8	2.62 ± 0.50	NR	NR	End of treatment (8w)	Mean score on the FOGQ reduced in the intervention group by 1.5 points, and in the control group by 0.07 points on average (p<0.01).
				C	NR	NR	13	NR	63.2 ± 3.3	6, 7	4.3 ± 0.7	2.69 ± 0.48	NR	NR		
Van Puymbroeck 2018[75]	Parallel groups	FOG-Q	3	A	Yoga	8w, 2pw, NR	15	NR	65.5 ± 6.1	10, 5	NR	2 ± 0.5	28.3 ± 14.9	NR	End of treatment (8w)	FOG reduced on average by 2.6 points after yoga (p=0.018, d=0.69) and 1.67 points (p=0.091, d=0.54) after wait-list control. No between group differences found.
				C	Passive: wait-list	NA	12	NR	70.5 ± 4.4	7, 5	NR	2 ± 1.0	31.6 ± 11.6	NR		
Carpinella 2017[76]	Parallel groups	FOG-Q	2	A	Physiotherapy with biofeedback (Gamepad system)	6w, 3pw, 20 sessions in total, 45min	17	NR	73.0 ± 7.1	14, 3	7.5 ± 3.2	2.7 ± 0.7	16.6 ± 6.8	NR	End of treatment (4-6w) and 1 month follow-up	No treatment effects found.
				C	Sham: physiotherapy without biofeedback	Same as A1	20	NR	75.6 ± 8.2	9, 11	10.3 ± 5.7	2.9 ± 0.5	22.3 ± 7.3	NR		
Carroll 2017[77]	Parallel groups	FOG-Q	2	A	Aquatic training	6w, 2pw, 45min	10	NR	69.5 (67.75 - 71.25)	7, 3	7 (3.25 - 12.3)	2.0 (1.5 - 2.25)	17.5 (8.75 - 21.3)	NR	End of treatment (6w)	No treatment effects found.
				C	Passive: usual care	NA	8	NR	74 (67 - 77)	5, 3	10.5 (4.25-13.5)	2.0 (1.63 - 2.88)	16.5 (10.3 - 21.3)	NR		
Santos 2017a[78]	Parallel groups	FOG-Q	2	A	Progressive resistance	8w, 2pw, 60-70min	13	NR	73.4 ± 8.8	5, 8	10.8 ± 4.1	1.92 ± 0.49	18.2 ± 12.1	458 ± 164	End of treatment (8w) and 1 month	No treatment effects found.

					exercise										follow-up	
				C	Passive: usual care	NA	15	NR	73.8 ± 7.1	10, 5	10.5 ± 4.0	1.86 ± 0.35	20.3 ± 11.8	474 ± 178		
Xiao 2017[79]	Parallel groups	FOG-Q	2	A	Tai-Chi ball exercises	12w, 4pw, 60min	25	NR	NR	NR	NR	NR	27.6 ± 4.34	NR	End of treatment (12w)	No treatment effects found.
				C	Passive: usual care	NA	25	NR	NR	NR	NR	NR	27.3 ± 5.16	NR		
Byl 2015[80]*	Parallel groups	FOG-Q	3	A	Gait training with visual and kinesthetic feedback on performance	6-8w, +-2pw, 12 sessions in total, 90min	7	NR	68.5 ± 3.6	4, 3	8.7 ± 4.4	NR	NR	NR	End of treatment (6-8w)	FOG is listed as an outcome, but data on FOGQ is not reported. Authors only mention subjects reported minimal changes in freezing (≤7%).
				C	Sham: gait training without feedback	Same as A1	5	NR	70 ± 2.9	4, 1	11.6 ± 5.9	NR	NR	NR		
Romenets 2015[81]	Parallel groups	FOG-Q	2	A	Tango	12w, 2pw, 60min	18	NR	63.2 ± 9.9	12, 6	5.5 ± 4.4	1.7 ± 0.6	20.7 ± 10.1	450 ± 350	End of treatment (12w)	No treatment effects found.
				C	Passive: wait list + pamphlet for PD exercises at home	NA	15	NR	64.3 ± 8.1	7, 8	7.7 ± 4.6	2.0 ± 0.5	27.5 ± 14.5	485 ± 348		
Duncan 2014[82]Δ	Parallel groups	FOG-Q	3	A	Tango	2years, 2pw, 60 min	5	NR	69.6 ± 6.6	4, 1	6.6 ± 7.5	2.4 ± 0.4	NR	NR	12 and 24 months after starting the treatment	No treatment effects found.
				C	Passive: usual care	NA	5	NR	66.0 ± 11	4, 1	11.0 ± 3.9	2.3 ± 0.3	NR	NR		
Paul 2014[83]	Parallel groups	NFOG-Q	2	A	Muscle power training	12w, 2pw, 45min	20	NR	68.1 ± 5.6	13, 7	7.8 ± 5.2	2.0 ± 0.7	37.1 ± 11.0	NR	End of treatment (12w)	No treatment effects found.
				C	Sham: low intensity exercises at home	12w, 2pw, unsupervised at home	20	NR	64.5 ± 7.4	12, 8	7.8 ± 5.9	1.9 ± 0.9	35.7 ± 14.0	NR		
Volpe 2013[84]	Parallel groups	FOG-Q	2	A1	Irish set dancing	24w, 1pw, 90min	12	NR	61.6 ± 4.5	7, 5	9.0 ± 3.6	2.2 ± 0.4	24.58 (3.87)	NR	End of treatment (24w)	Irish set dancing significantly improved FOG compared to physiotherapy, which did not improve FOG.
				A2	Conventional physiotherapy including cueing that was not aimed at FOG	Same as A1	12	NR	65.0 ± 5.3	6, 6	8.9 ± 2.5	2.2 ± 0.4	23.92 (3.50)	NR		
Duncan 2012[85]**	Parallel groups	FOG-Q	2	A	Tango	1year, 2pw, 60min	26	NR	69.3 (1.9)#	15, 11	5.8 (1.1)#	2.6 (0.1) #	44.5 (2.3)# (during OFF)	NR	3, 6 and 12 months after starting the treatment	No between group differences at any time point, but a significant group x time interaction with the control group scoring worse after 12 months, while the Tango group did not change over time.
				C	Passive: usual care	NA	26	NR	69.0 (1.5)#	15, 11	7.0 (1.0)#	2.5 (0.1) #	48.0 (1.8)#	NR		
Reuter 2011[86]	Parallel groups	UPDRS-III FOG item	3	A1	Nordic walking	24w, 3pw, 70min	30	NR	62 ± 3.2	NR	5.34 ± 4.1	2.47 ± 0.51	NR	NR	End of treatment (24w)	40% of Nordic walking, 36.7% of regular walking, and 10% of patients in the flexibility

				A2	Walking	Same as A1	30	NR	63 ± 3.1	NR	6.0 ± 4.2	2.50 ± 0.51	NR	NR		and relaxation training reduced FOG post-intervention by 2 points, but group and time effects are not reported.
				A3	Flexibility & relaxation	Same as A1	30	NR	62.1 ± 2.5	NR	5.19 ± 3.2	2.53 ± 0.51	NR	NR		
Hackney 2009[87]	Parallel groups	FOG-Q	3	A1	Tango	13w, 20 sessions, 60min	14	57.1	68.2 (1.4)#	11, 3	6.9 (1.3)#	2.1 (0.1)#	27.6 (2.0)#	NR	End of treatment (13w)	No treatment effects found.
				A2	Waltz/ Foxtrot	Same as A1	17	52.9	66.8 (2.4)#	11, 6	9.2 (1.5)#	2.0 (0.2)#	26.9 (2.5)#	NR		
				C	Passive: usual care	NA	17	29.4	66.5 (2.8)#	12, 5	5.9 (1.0)#	2.2 (0.2)#	27.4 (2.4)#	NR		
Hackney 2007[88]	Parallel groups	FOG-Q	3	A	Tango	13w, 20 sessions, 60min	9	NR	72.6 (2.2)#	6, 3	6.2 (1.5) #	2.3 (0.7)#	30.6 ± 1.3	NR	End of treatment (13w)	Significant main effect of time for the sample as a whole (p=0.044), but no significant change within groups.
				C	Sham: strength and flexibility exercises	Same as A	10	NR	69.6 (2.1) #	6, 4	3.3 (0.5) #	2.2 (0.6)#	28.2 ± 1.2	NR		
Pacchetti 2000[89]	Parallel groups	UPDRS-II FOG item	3	A1	Music therapy (i.e. use of musical instruments)	12w, 1pw, 120min	16	NR	62.5 ± 5	12, 4	4.8 ± 3	NR	40.2 ± 7.7	NR	1, 3, 5, 7, 9, 11 weeks after starting the treatment and 2 months follow-up	Post-hoc analyses revealed significant changes in self-reported freezing after music therapy, but the data is not presented.
				A2	Conventional physiotherapy including gait and balance	12w, 1pw, 90min	16	NR	63.2 ± 5	11, 5	5.2 ± 2	NR	40.7 ± 7	NR		

Footnote: Values presented as (Mean ± SD) or (Median (Interquartile Range)) or #=Mean (Standard error) or $=Median (Range). AO=Action observation; FOG=Freezing of gait; (N)FOGQ= (New) Freezing of gait questionnaire; FOG score = severity of FOG scored during FOG-provoking gait assessment as per Ziegler et al. (2010); FOG-ratio=objective measure of FOG severity derived from inertial measurement units during turning on the spot; Outcome=Outcomes of the study that were directly related to FOG severity; FOG Obj.= FOG Objective, i.e. whether FOG was considered the Primary (1), Secondary (2) or Tertiary (3) outcome of the trial; Groups: A=Active Intervention group, C=Control group; Dosage of intervention=Number of weeks (w), number of sessions per week (pw), and duration of each session in minutes (min); N=Number of subjects analyzed in each group for the FOG outcome at the primary endpoint; %FOG = Percentage of subjects that were classified as being 'freezers' (i.e. people with Parkinson's disease who experience FOG) at baseline; Age in years; DD=Disease duration (time since diagnosis) in years; HY=Hoehn and Yahr scale; UPDRS-III=Unified Parkinson's Disease Rating Scale Motor Section, part III; LEDD=Levodopa Equivalence Daily Dose; NA = Not applicable; NR = Not Reported or values could not be computed with the information provided. * Authors contacted by email to retrieve FOG data for inclusion in the meta-analysis; **FOG meta-data provided by authors upon email request, Δ=Same participants as Duncan et al. (2012).

Supplementary Data 1 - Funnel Plots

1. Primary analysis

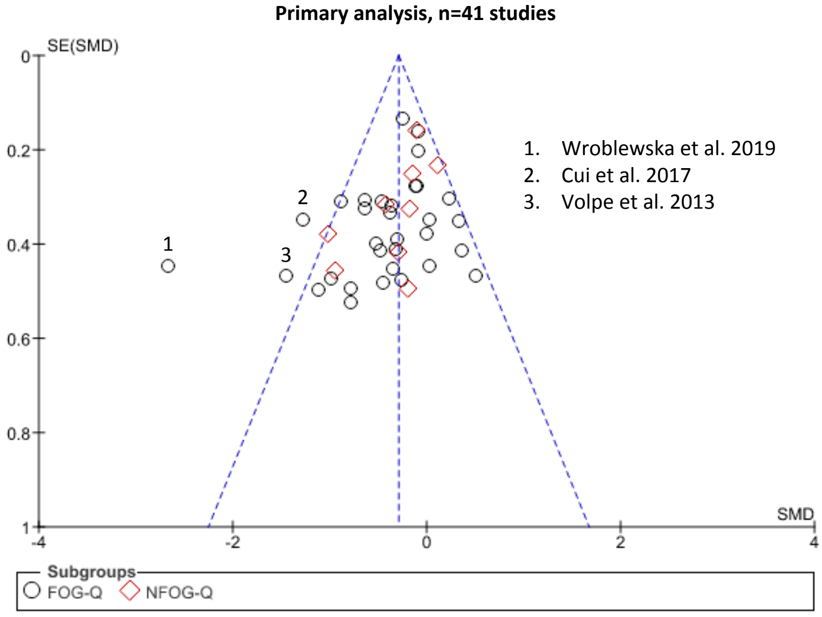

2. Secondary analyses

2.1 Passive control groups

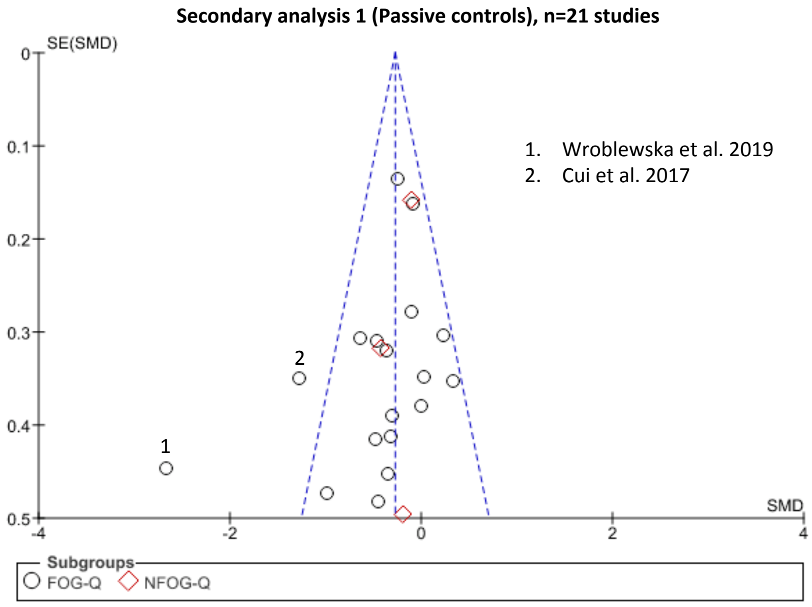

2.2 Active control groups

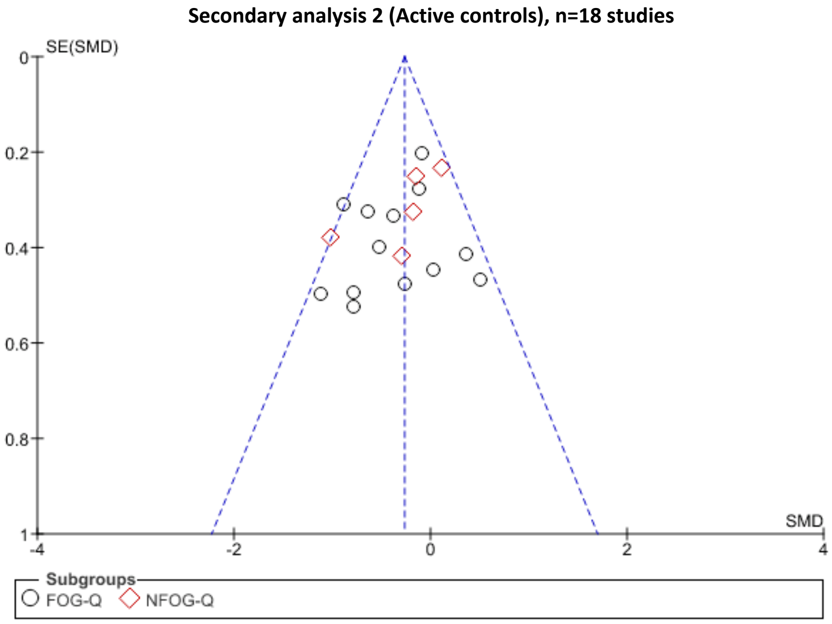

2.3 Freezers only

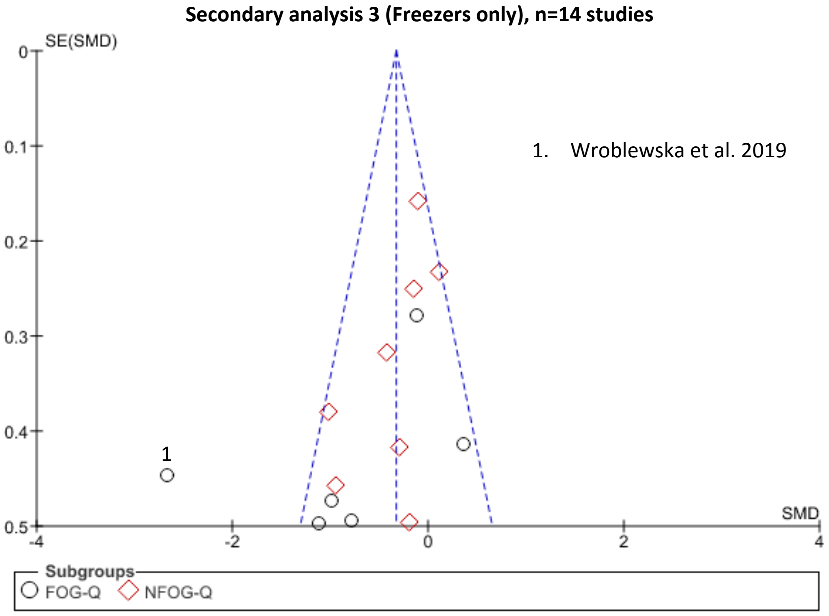

2.4 Subtype, Category A (FOG-specific)

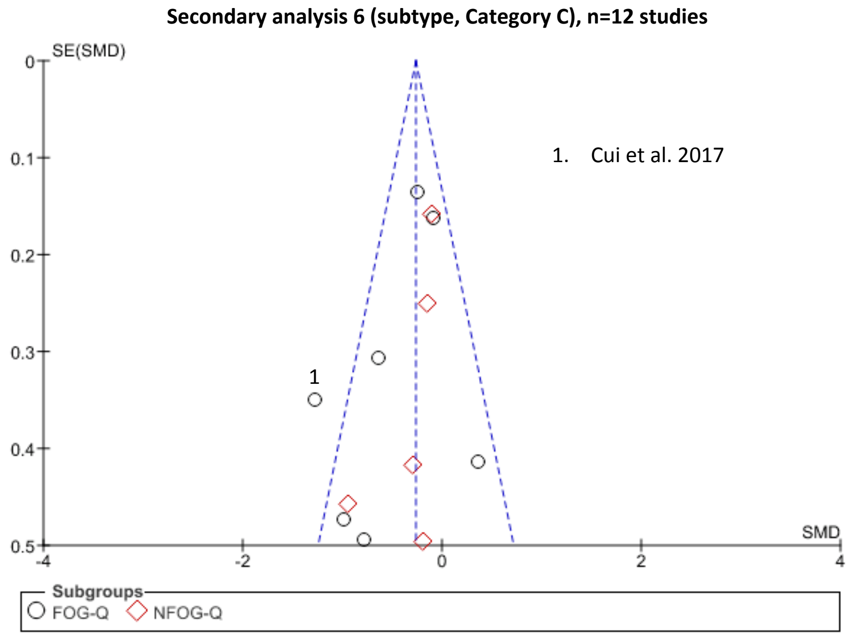

2.5 Subtype, Category B (FOG-relevant)

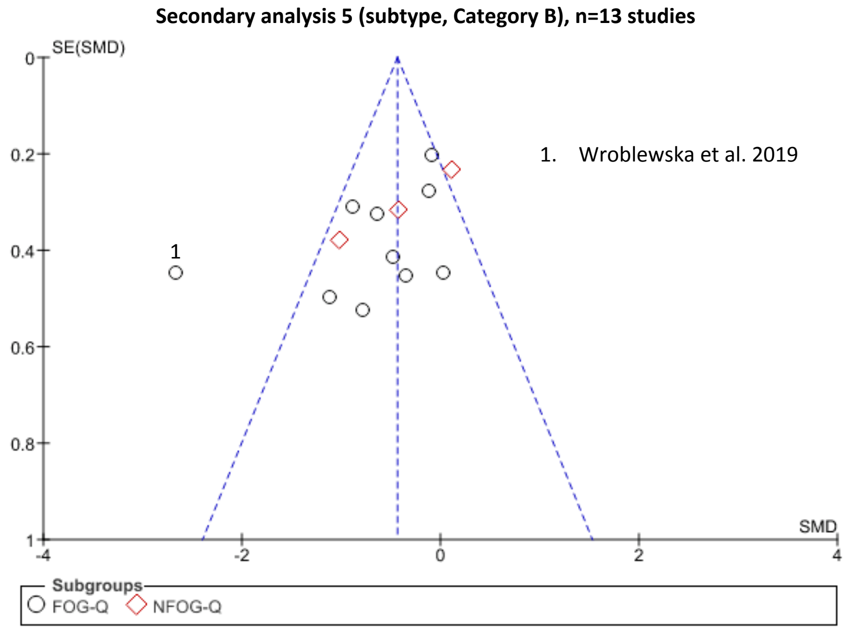

2.6 Subtype, Category C (generic exercise)

Secondary analysis 4 (subtype, Category A), n=16 studies

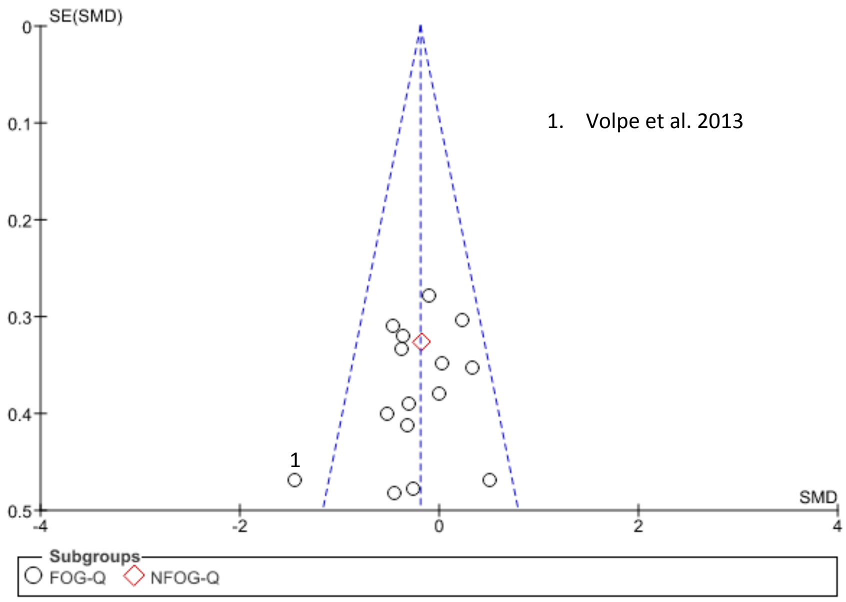

2.7 Retention

Secondary analysis 7 (Retention), n=15 studies

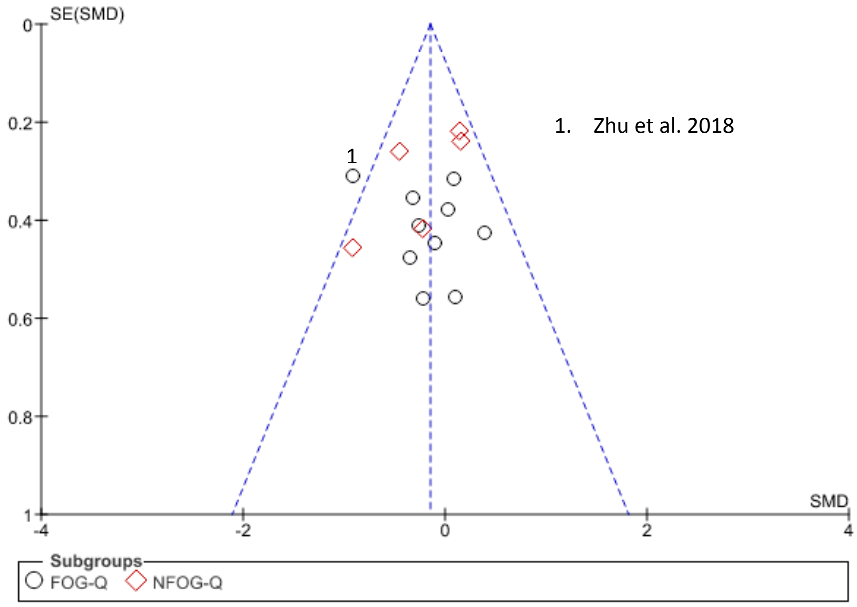

Supplement: Supplementary file 1 — Supplementary Information [file 41531_2021_224_MOESM1_ESM.pdf]
